# HaloTag-based conjugation of proteins to barcoding-oligonucleotides

**DOI:** 10.1093/nar/gkz1086

**Published:** 2019-11-22

**Authors:** Junshi Yazaki, Yusuke Kawashima, Taisaku Ogawa, Atsuo Kobayashi, Mayu Okoshi, Takashi Watanabe, Suguru Yoshida, Isao Kii, Shohei Egami, Masayuki Amagai, Takamitsu Hosoya, Katsuyuki Shiroguchi, Osamu Ohara

**Affiliations:** 1 Laboratory for Integrative Genomics, RIKEN Center for Integrative Medical Sciences (IMS), Yokohama City 230-0045, Japan; 2 Laboratory for Prediction of Cell Systems Dynamics, RIKEN Center for Biosystems Dynamics Research (BDR), Osaka 565–0874, Japan; 3 Laboratory of Chemical Bioscience, Institute of Biomaterials and Bioengineering, Tokyo Medical and Dental University, Tokyo 101-0062, Japan; 4 Common Facilities Unit, Compass to Healthy Life Research Complex Program, RIKEN Cluster for Science, Technology and Innovation Hub, Kobe 650-0047, Japan; 5 Laboratory for Skin Homeostasis, RIKEN Center for Integrative Medical Sciences (IMS), Yokohama 230-0045, Japan; 6 Department of Dermatology, Keio University School of Medicine, Tokyo 160–8582, Japan; 7 Laboratory for Chemical Biology, RIKEN Center for Biosystems Dynamics Research (BDR), Kobe 650-0047, Japan; 8 Laboratory for Immunogenetics, RIKEN Center for Integrative Medical Sciences (IMS), Yokohama 230-0045, Japan

## Abstract

Highly sensitive protein quantification enables the detection of a small number of protein molecules that serve as markers/triggers for various biological phenomena, such as cancer. Here, we describe the development of a highly sensitive protein quantification system called HaloTag protein barcoding. The method involves covalent linking of a target protein to a unique molecule counting oligonucleotide at a 1:1 conjugation ratio based on an azido–cycloalkyne click reaction. The sensitivity of the HaloTag-based barcoding was remarkably higher than that of a conventional luciferase assay. The HaloTag system was successfully validated by analyzing a set of protein-protein interactions, with the identification rate of 44% protein interactions between positive reference pairs reported in the literature. Desmoglein 3, the target antigen of pemphigus vulgaris, an IgG-mediated autoimmune blistering disease, was used in a HaloTag protein barcode assay to detect the anti-DSG3 antibody. The dynamic range of the assay was over 10^4^-times wider than that of a conventional enzyme-linked immunosorbent assay (ELISA). The technology was used to detect anti-DSG3 antibody in patient samples with much higher sensitivity compared to conventional ELISA. Our detection system, with its superior sensitivity, enables earlier detection of diseases possibly allowing the initiation of care/treatment at an early disease stage.

## INTRODUCTION

Protein profiling is a major strategy used in post-transcriptome assays to assign a function to uncharacterized protein-coding genes. It is important not only for gene characterization in basic biological studies but also for medical diagnosis, e.g. for antibody-based assays of an immune system disorder, such as autoimmune diseases. Approaches based on physical protein interactions include enzyme-linked immunosorbent assay (ELISA), protein microarrays, affinity purification-mass spectrometry, and yeast two-hybrid system. These approaches are used to characterize cellular signaling networks and facilitate candidate biomarker discovery (1–3). Conventional protein profiling technologies involve the use of such dedicated platforms as a mass spectrometer or microarray platform (4–12). Next-generation sequencing (NGS) for investigating genome dynamics has rapidly emerged in the last decade. It is widely available and indispensable technology worldwide. Protein profiling involving NGS has been used to identify target protein molecules, e.g. for protein–protein interaction (PPI) analysis and antibody-transcriptome profiling (13–16). NGS technologies not only increase the number of target molecules that can be assayed at any time but also facilitate detection of target molecules present in low copies because of the nucleic acid amplification involved, regardless of the observed amplification bias (17). However, to address the NGS-associated amplification bias, multiplexed molecular barcoding methods that minimize the bias have been proposed (17–19). Protein conjugation to DNA molecules is increasingly used for antibody labeling (4,13,15), proximity ligation (20,21), and cell imaging (22,23). Generally, the target protein is conjugated to another molecule (DNA or a fluorophore) modified by an activated ester, such as *N*-hydroxysuccinimide, via the amino group of the target protein. The formation of an ester-amine by covalent bonding in a pH-dependent manner can be used to modify proteins *in vivo* (24,25). On the other hand, conjugation reaction via click chemistry is rapidly emerging for many organic reactions in the biological field because of several advantages, such as pH-insensitivity and reactivity in water with no apparent toxicity (26,27). Here, we report the development of a protein–oligonucleotide conjugation method involving a high-affinity capture tag, HaloTag, to link proteins to DNA oligonucleotides, and its application in protein profiling, including antigen–antibody interactions.

## MATERIALS AND METHODS

### Preparation of a barcoded HaloTag protein complex

**Figure 1.**
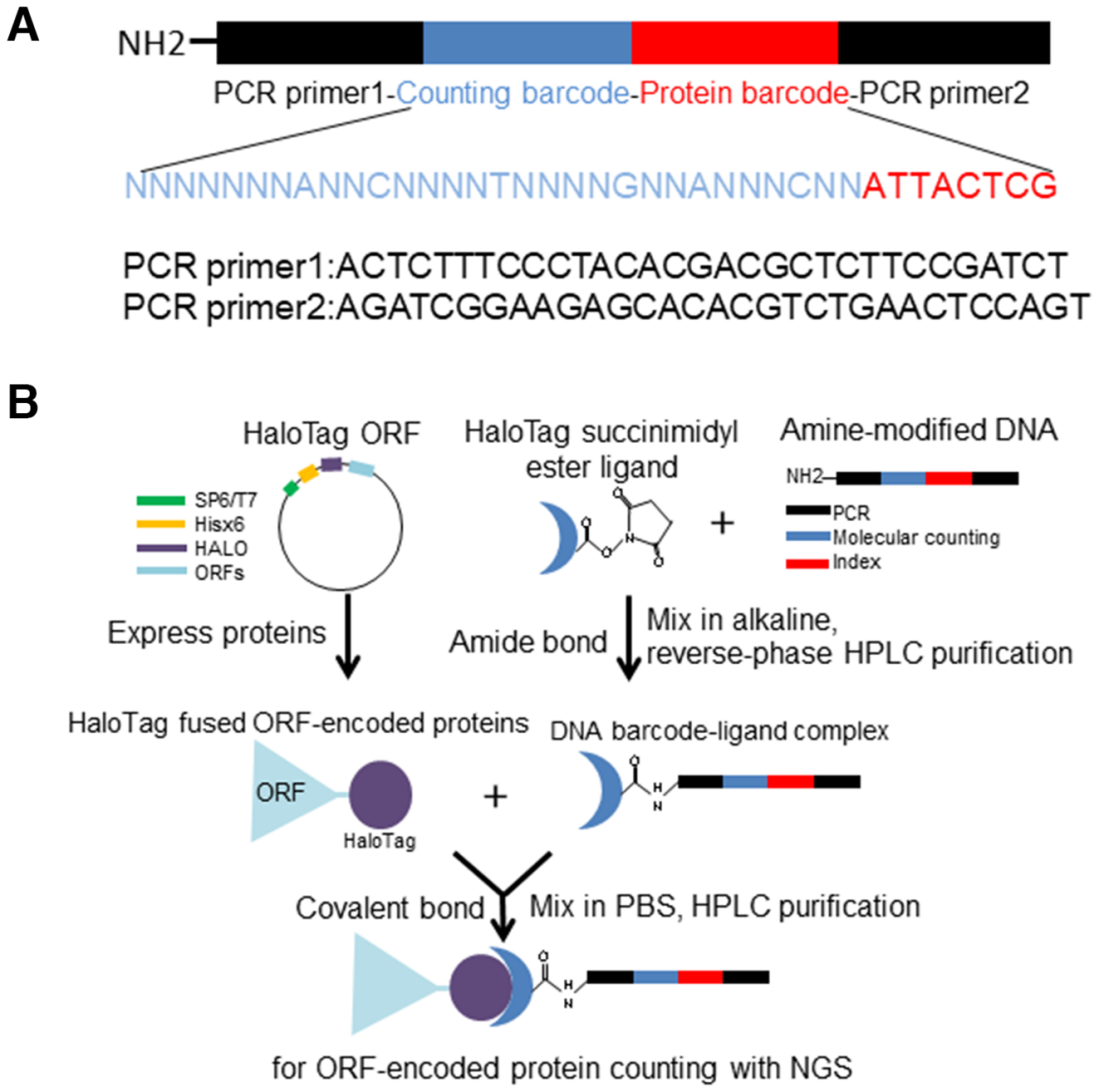
HaloTag-based protein barcoding. (**A**) Schematic diagram of the amine-modified barcode DNA. (**B**) The HaloTag-based protein barcoding assay. Plasmids harboring specific ORFs fused with HaloTag are first prepared. Addition of a coupled transcription-translation reagent results in the expression of a HaloTag fusion ORF protein. The HaloTag protein binds covalently to ester–chloroalkane ligand linked to an amido-modified oligonucleotide. The method enables protein molecule detection using the DNA barcode.

The initial preparation of the HaloTag-barcoded-protein was performed using a first-generation set of custom proteins, HaloTag protein G (1 μg/μl, Kazusa DNA Research Institute, Japan), NanoLuc-HaloTag (8 μg/μl, NL-HaloTag; Promega, USA), HaloTag-FOS proto-oncogene proteins (40 ng/μl, HaloTag-FOS; Cell Free Science, Japan), and HaloTag-Glutathione S-transferase (3 μg/μl, HaloTag-GST; Promega). DNA encoding the protein identifier to identify the protein type (Figure 1A: red, 8 bp; and Supplementary Table S1), semi-random bases for molecule counting (Figure 1A: blue, 30 bp; and Supplementary Table S1), and the amplification base for polymerase chain reaction (PCR) reaction (Figure 1A: black, 31 bp, 2×; and Supplementary Table S1) were prepared with amine modification by *N*-hydroxysuccinimide at 5′-end of the barcode DNA (Figure 1A, Eurofins, Japan). The HaloTag ligand–oligonucleotide complex was formed using the amido bond-based method (28). Briefly, 300 μl 100 μM DNA was mixed with 90 μl 0.9 M sodium bicarbonate solution and 20 μl 50 mM HaloTag O4 ester ligand (Promega), and incubated at room temperature (RT, 25°C) for 1 h. The formed complex was subsequently purified by ethanol precipitation and NAP-5 gel filtration (GE Healthcare, USA), followed by reversed-phase high-performance chromatography (HPLC; Shimadzu, Japan), and eluted using an acetonitrile gradient. To create the DNA-HaloTag fusion protein complex, 5 μg HaloTag fusion protein was mixed with 2 μg ligand–oligonucleotide complex, in phosphate-buffered saline buffer containing 0.05% (vol/vol) Nonidet P-40 (PBS-NP40), and incubated for 1 h at RT before subsequent purification by cation-exchange HPLC (Shimadzu).

### Protein quantification and pull-down using barcoded proteins

For immunoglobulin G (IgG)-protein G PPI assay, 75 μl of each bait protein, DNA-barcoded HaloTag protein G [93 nM, as determined by quantitative PCR (qPCR), see below] and HaloTag-GST (35 nM, as determined by qPCR) in PBS–NP40 were mixed with 10 μl mouse IgG magnet beads (Thermo Fisher, USA), according to the manufacturer's instructions, and incubated at 4°C for 2 h. The mixture was then washed three times with 500 μl PBS-NP40 and the washed beads were boiled in 100 μl PBS-NP40 at 90°C for 5 min. The barcoded proteins in the boiled mixture were quantified using qPCR, which was performed using the LightCycler 480 (Roche, Switzerland) and KAPA SYBR FAST (Sigma Aldrich, USA). For each reaction, standard curves for each DNA barcode were constructed using 5 serial dilutions of the DNA barcode. The relative amount of the barcode DNA was calculated by comparing with the standard curves. Specific primers (primer 1: 5′- ACACTCTTTCCCTACACGACGCTCTTCCGATCT-3′; and primer 2: 5′-GTGACTGGAGTTCAGACGTGTGCTCTTCCGATCT-3′) for qPCR were designed for the following conditions: 5 min at 95°C; followed by ∼35 cycles of 30 s at 95°C and 45 s at 60°C; 100-bp products were thus generated in triplicate. For the well-known JUN-FOS PPI assay (29), the sequence encoding a hexa histidine-tagged (6× His-tag) HaloTag-JUN was cloned into pEU-E01-MCS vector (Cell Free Science) and the protein synthesized *in situ* from plasmid DNA using the SP6 TNT wheat germ system (Promega), according to the manufacturer's recommendations. Then, 25 μl of the bait protein (HaloTag-JUN) was mixed by rotating with 10 μl Halo magnetic beads (Promega) in a total volume of 100 μl in PBS–NP40 at RT for 1 h. Subsequently, beads with the HaloTag-JUN fusion protein were washed and added to 25 μl of the prey protein (barcoded HaloTag-FOS, 25 nM, or HaloTag protein only, 50 nM, as determined by qPCR), and then mixed by rotating at 4°C for 2 h. The formed complex was then washed three times with 500 μl PBS-NP40 and the washed beads were boiled in 100 μl PBS-NP40 at 95°C for 5 min. The barcoded proteins in the boiled mixture were quantified by using qPCR, as described above.

### Detection of the NanoLuc fusion-barcoded proteins

The sensitivity of the barcoding assay was evaluated by determining the number of DNA oligonucleotides on the barcoded proteins. Dilution series of the barcoded proteins were prepared, and DNA was amplified using a set of specific primer combinations and by indexing (Supplementary Table S2), using Mighty Amp DNA polymerase PCR (Takara, Japan). The barcoded DNA template was PCR-amplified as follows: 2 min at 98°C; followed by 4 cycles of 10 s at 98°C, 10 s at 60°C, and 1 min at 68°C; 25 cycles of 10 s at 98°C, 2 s at 60°C and 1 min at 68°C; and a final extension step of 5 min at 68°C. The amplified DNA was purified by using the Agencourt AMPure XP kit (Beckman, USA), and the quality of the DNA library was assessed by using a Bioanalyzer (Agilent, USA), as recommended by the manufacturer. The amplified 174-bp products, including the Illumina adapter and index sequence, were analyzed using the MiSeq system (Illumina, USA) with dual-indexed sequencing in a paired-end flow cell, to read 76 bp, including the amplified 38 bp of the barcode DNA (Figure 1A, blue and red, and Supplementary Table S1). After sequencing various mixed fusion protein libraries, each protein was identified by the 8-bp protein barcode sequence (Figure 1A, red, and Supplementary Table S1) using Bowtie2 v.2.2.9 (30). The proteins were then quantified using the 30-bp sequence tag containing 24 random bases and 6 fixed bases (Figure 1A, blue, and Supplementary Table S1). Barcode-based protein molecule counting methods were as described elsewhere, using the parameters: *Distance*, 2; *fixed base number*, 6; and *random base number*, 24 (18). Briefly, the counting barcode region (Figure 1A, blue, and Supplementary Table S1), a 30-bp sequence tag, was used to determine the molecule number after identifying each protein based on the 8-bp protein barcode sequence (Figure 1A, red, and Supplementary Table S1). The fixed 6 bases in the counting barcode [A, C, T, G, A and C in Figure 1A (blue) and Supplementary Table S1 (Oligo01)] were used for filtering to exclude molecules with at least one mismatched fixed base from the molecule counting process. Following the filtering, an in-house clustering software ‘Nucleotide Sequence Clusterizer’ was used for barcode clustering to determine the number of protein molecules (18).

NanoLuc luciferase assays were performed in triplicate experiments using the Nano-Glo luciferase assay system (Promega), as recommended by the manufacturer. Briefly, 100 μl of serially diluted barcoded proteins (10^3^–10^7^ dilution) in PBS was added to wells of a black micro-well plate (used to avoid signal dispersion). The substrate was then added, and in-well luminescence was determined by using the Fusion alpha plate reader at 460 nm (PerkinElmer, USA).

### Construction of click chemistry-based HaloTag-barcoded protein

A new click chemistry-based labeling technique was developed to generate HaloTag-barcoded proteins. The developed click chemistry-based protocols eliminate the cumbersome process of complex preparation involving the amido-bond-based method. To develop the new method, 2 types of azido-HaloTag ligand, AzHLT-1 (31) and AzHLT-2 (Supplementary Method andSupplementary Figure S1A), were used in a strain-promoted click reaction with cycloalkyne-modified 100-bp DNA oligonucleotides. For the HaloTag ligand–oligonucleotide complex formation by click labeling, 2 μl 500 μM DNA 5′-modified by dibenzocyclooctyne (DBCO) (Supplementary Table S1; Eurofins) was mixed with 0.5 μl 500 μM azido-HaloTag ligand in water (4:1), and incubated at RT for 1 h (click reaction). The complex was then used to directly create a DNA–HaloTag fusion protein complex. Specifically, 1–2 μg HaloTag fusion proteins were mixed with 1 μl of the ligand–oligonucleotide complex, and incubated at RT for 1 h. The DNA–HaloTag fusion protein complex was then purified by histidine tag purification (Mettler Toledo, USA) to eliminate unbound DNA, azido-HaloTag ligand and the HaloTag ligand–oligonucleotide complex. Since the preparation of the HaloTag-barcoded proteins is very simple and the AzHTL-1 ligand captured more protein than the AzHTL-2 ligand, the AzHTL-1 ligand was used for the preparation of all proteins by HaloTag barcoding for the subsequent interaction assay.

### Vector construction

Vector pIX-His-HALO was constructed by inserting a sequence encoding a 6× His-tag by PCR using the histidine cassette primers (primer 1: 5′-GTGATGATGCATGATATCTGTAGTTGTAGAATGT-3′; and primer 2: 5′-CATCACCACGCAGAAATCGGTACTGGCTTTCCAT-3′) downstream of the T7 promoter on the pIX-Halo:ccdB vector (11; https://www.arabidopsis.org/servlets/TairObject?type=vector&id=1001200298). This was followed by self-ligation of primers and the 3× His-tag–encoding sequences.

### PPI assay with a reference set of HaloTag-barcoded proteins

The used reference set of proteins consisted of a randomly picked subset of proteins described previously (11). The PPI assays were performed using Magne HaloTag beads (Promega), following the manufacturer's recommendations. The open reading frames (ORFs) encoding the reference protein set were inserted into vector pIX-His-Halo:ccdB (11) using the Gateway LR recombination cloning system (Invitrogen, USA). The LR recombination products were then used to transform competent *Escherichia coli* DH5α-T1R cells. Single colonies of the transformants were picked from LB agarose medium (32) containing 50 μg/ml ampicillin, the clones were cultured in 5 ml liquid terrific broth medium (33) overnight at 37°C, and plasmid DNA was purified using a DNA purification kit (Qiagen, Germany). The proteins were expressed from pIX-His-Halo vectors using a T7 TNT-coupled wheat germ extract system for protein expression (Promega), according to the manufacturer's instructions. HaloTag-fused proteins were then labeled with a Halo ligand–oligonucleotide complex prepared by click chemistry. Briefly, 150 μl synthesized proteins (unpurified reference protein set) was mixed with 1 μl ligand–oligonucleotide complex in 5 mM EDTA, and incubated at RT for 1 h. This was followed by histidine tag column purification (Mettler Toledo) and dialysis into PBS buffer using an Amicon-ultra 10k column (Merck-Millipore, Germany). The barcoded proteins were treated with HaloTag ligand (Promega) at RT for 1 h to fill the empty HaloTag ligand capture sites; 25 μl bait protein (pIX-His-HALO-ORFs) produced using the TNT-coupled wheat germ extract (Promega) was mixed with 5 μl Magne HaloTag beads and incubated at RT for 1 h. The beads coupled with bait proteins were then washed with PBS and mixed with 5 μl barcoded proteins at RT for 2 h. The mixture was next washed 3 times with PBS and used for library preparation for NGS. The barcode count that exceeded background signal intensity (the number of sequence reads) from barcoded HaloTag-only protein (the negative control) was considered to be positive. The barcoded proteins whose reads were <10 were considered as negative pairs of PPI (Supplementary Table S3). For high-throughput assay of a barcoded 51 protein mixture, 10 μl of each barcoded protein was mixed, purified using a histidine tag column (Mettler Toledo), and dialyzed into PBS buffer using an Amicon-ultra 10k column (Merck-Millipore) (Supplementary Table S4). Then, 9 μl bait protein (pIX-His-HALO-bZIP53) produced using the TNT-coupled wheat germ extract (Promega) was mixed with 5 μl Magne HaloTag beads and incubated at RT for 1 h. The beads coupled with bZIP53 bait proteins (AT3G62420, https://www.arabidopsis.org/) were then washed with PBS and mixed with 1 μl of a barcoded 51 protein mixture at RT for 2 h. Finally, the mixture was washed three times with PBS and used for library preparation for NGS.

### Desmoglein 3 (DSG3) barcode immunoprecipitation

The extracellular domain of calcium-dependent protein DSG3 (34, pEVmod-Dsg3-His) and HaloTag from pIX-His-HALO vector were cloned by overlapping PCR using the following primers: primers for DSG3; Dsg3L: 5′-ATGATGGGGCTCTTCCCCAGAAC-3′, Dsg3R: 5′-ATCCTCCTCCTTGGAAGTACAGGTTTTCGTGCACCCTCCCTGAGTGCGGCC-3′, primers for HaloTag; HaloL: 5′-ACCTGTACTTCCAAGGAGGATCCGAAATCGGTACTGGCTTTCC-3′

HaloR: 5′-TCAATGATGATGATGATGATGACCGGAAATCTCCAGAGTAGACAGC-3′. Halotag-DSG3-extracellular domain construct was inserted into pcDNA3.4 TOPO vector by pcDNA3.4 TOPO TA cloning kit (Thermo Fisher) according to the manufacturer's protocol. HaloTag-DSG3 extracellular domain clones were used to transform into Expi293F cells (Gibco, USA) using the Expi293 expression system (Thermo Fisher) in an expression medium for 5 days as per manufacturer's instructions with d-PBS(+) preparation reagent (Ca, Mg solution) (Nacalai tesque, Japan). The proteins were purified from the supernatant using a His-tag purification kit (Mettler Toledo). The proteins were barcoded using 100-bp oligonucleotides (Oligo59–62, Supplementary Table 1), as described above, as ligand-oligonucleotide complexes conjugated through click reaction. Dilution series (10^4^–10^18^ dilution) of anti-DSG3 monoclonal antibody (35) (Medical & Biological Laboratories, Japan) were prepared and 25 μl of each dilution was mixed by rotating with 10 μl protein G beads (Thermo Fisher) in Tris-buffered saline with 1 mM calcium chloride (36, TBS-Ca) at RT for 1 h. The beads with an anti-DSG3 antibody were washed three times with 75 μl TBS-Ca. Then, 1 μl barcoded HaloTag-DSG3 protein was added to the beads and rotation-mixed at 4°C for 2 h. The barcoded proteins in the mixture were washed three times with 75 μl TBS-Ca. The mixture of beads and barcoded proteins was used for qPCR and sequencing using the Illumina MiSeq system, as described above. The reads of barcoded DSG3 were determined based on the dilution series of anti-DSG3 antibody used in each immunoprecipitation reaction. The barcoded immunoprecipitation reactions whose sum of barcode Oligo59–62 reads was greater than that of the background control (barcoded HaloTag protein only with Oligo71–74 or Oligo75–78, Supplementary Table S1) were considered as positive. The read numbers of positive reactions were then divided according to the read number after immunoprecipitation with the human serum (Sigma Aldrich) to determine the specificity. For validating the clinical application of the system, dilution series (10^4^ or 10^6^ dilutions) of 4 human serum samples (2 patients diagnosed with pemphigus vulgaris (PV) and 2 healthy controls) were prepared and 20 μl of each dilution was mixed with 20 μl of protein G beads (Thermo Fisher) in Tris-buffered saline with 1 mM calcium chloride (36, TBS-Ca) by rotating at RT for 1 h. The beads with IgG were washed 3 times with 75 μl TBS-Ca and the rest of the assay was performed as described above. All 4 clinical specimens were obtained from Keio University Hospital (Tokyo, Japan) where a chemiluminescent enzyme immunoassay was performed for the diagnosis of PV. All study participants provided signed informed consent, and the study protocol was approved by the Institutional Review Boards of the RIKEN Center for Medical Sciences.

### DSG3 ELISA immunoprecipitation

ELISA assay for the anti-DSG3 antibody was performed in triplicate by using MESACUP2 for DSG3 (Medical & Biological Laboratories), according to the manufacturer's recommendations. Briefly, 100 μl of serial anti-DSG3 monoclonal antibody dilutions (10^2^–10^8^ dilutions) was added to DSG3-coated wells and incubated for 1 h at RT. Wells containing the primary anti-DSG3 antibody were then washed with 100 μl TBS-Ca 3 times and incubated with an anti-mouse secondary polyclonal antibody coupled to horseradish peroxidase (HRP) [1:1000 (vol/vol), Cell Signaling, USA] for 1 h at RT. The wells were washed with TBS-Ca before the addition of the HRP substrate from the kit (Medical & Biological Laboratories), according to the manufacturer's recommendations. HRP concentration was determined based on the absorbance at 450 nm using a GloMax plate scanner (Promega). For the clinical samples, 100 μl of serial serum dilutions (10^2^–10^4^ dilutions) was added to MESACUP2 DSG3-coated wells and incubated for 1 h at RT (Medical & Biological Laboratories). The rest of the assay was performed using the MESACUP2 for DSG3 kit (Medical & Biological Laboratories), following the manufacturer's recommendations.

HaloLink plate assay with the anti-DSG3 antibody was performed using HaloLink 96-well white bottom plates (Promega), following the manufacturer's instructions. For the assay, 50 μl of serially diluted antigen (HaloTag-DSG3; 0.0001–400 ng) in TBS-Ca, to avoid deactivation of the DSG3 protein, was added to HaloTag ligand-coated wells, and incubated for 2 h at RT. After three washes with TBS-Ca, the anti-DSG3 antibody was added and incubated with the HaloTag-DSG3 protein captured in the wells for 1 h at RT. The wells containing the primary antibody were then washed with TBS-Ca buffer and incubated with anti-mouse HRP-coupled secondary antibody [1:1000 (vol/vol), Cell Signaling] for 1 h at RT. The wells were then washed with TBS-Ca buffer before the addition of the HRP substrate (ECL western blotting detection reagents; GE Healthcare), according to the manufacturer's recommendations. Sample chemiluminescence was detected using the GloMax plate scanner (Promega). The average signal intensity was calculated based on data from three replicate wells. The readings whose intensity was higher than one standard deviation (SD) over that of the negative control average (no anti-DSG3 antibody) were considered to be positive.

### Statistical analysis

Two-tailed Fisher's exact test was performed in QuickCalcs at GraphPad (http: https://www.graphpad.com/quickcalcs/contingency1/). A *P*-value smaller than 0.05 was considered statistically significant.

## RESULTS

### Development of a HaloTag-based protein barcoding assay

**Figure 2.**
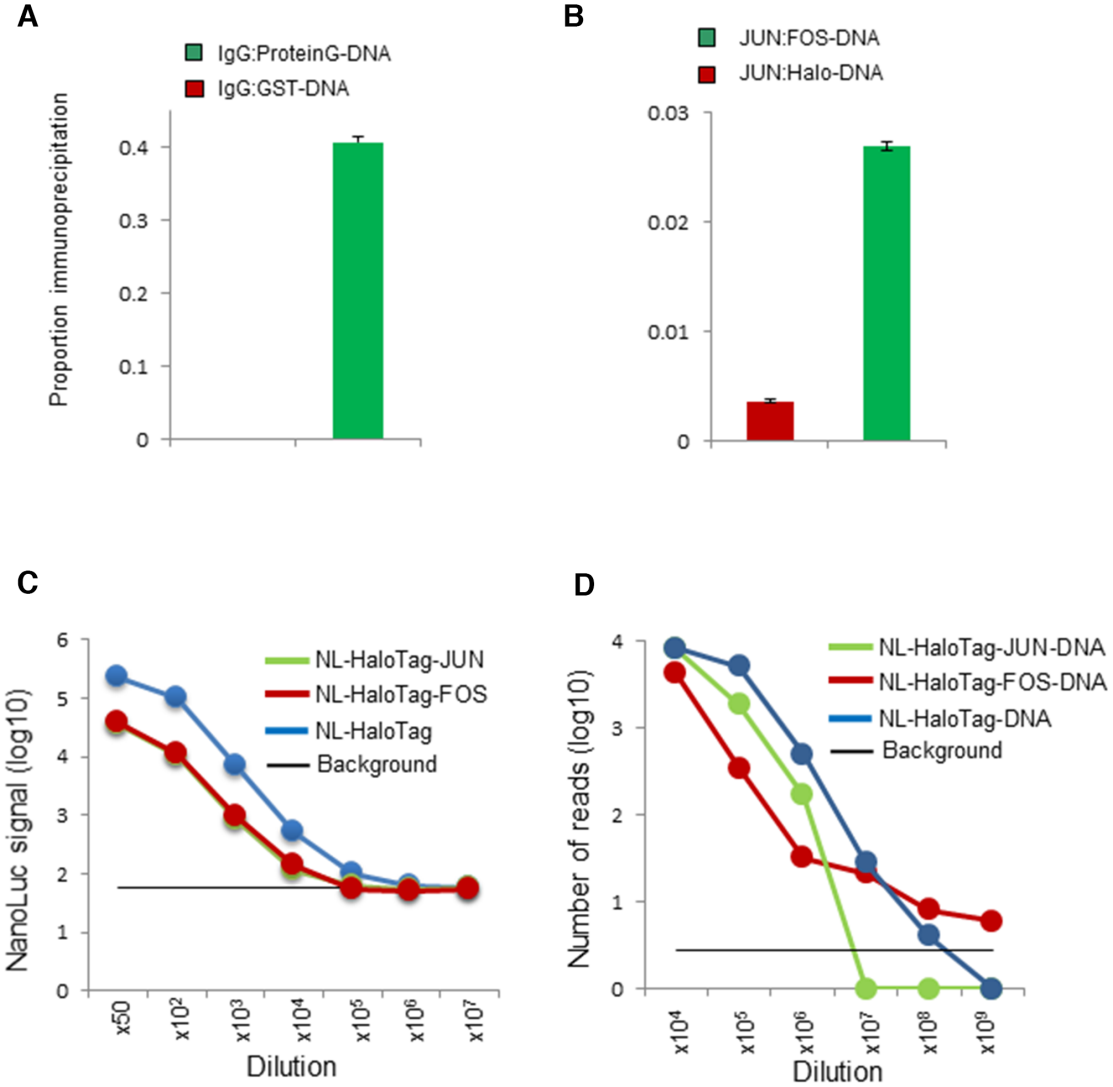
The HaloTag barcoding assay allows visualization of a clear interaction signal of an active protein. (**A**) The interaction between barcoded protein G and IgG, as detected by qPCR produced a signal that was 1000-fold greater than that from the negative control interaction between barcoded GST and IgG. (**B**) Protein interaction between barcoded FOS and JUN proteins produced a signal that was seven times greater than that from the negative control interaction pair of barcoded HaloTag and JUN. The proportion of specific reads of the DNA barcode after pull-down experiments are shown on the y-axis. (**C**) Direct comparison of the HaloTag barcoding assay and luciferase assay. The sensitivity of the luciferase assay determined using 3 different proteins [JUN (green), FOS (red), and HaloTag (blue)] is shown. The background signal (black) indicates the average signal from negative control (water) + 3 SD. (**D**) The sensitivity of the HaloTag barcoding assay determined using three different fusion proteins [JUN (green), FOS, (red) and HaloTag (blue)]. The background signal (black) indicates the number of reads from the DNA barcode sequence that are unmapped (not used in the assay).

Aqueous solution-based protein assaying technology is unparalleled with respect to its ability to interrogate thousands of correctly folded proteins in a single experiment using a wide variety of probes (37,38). A solution-based technology, e.g. protein complex purification coupled with mass spectrometry, plays an important role in assay biochemistry, as it is not limited by the need to anchor the target protein to a solid support, as in, e.g. protein array, which may limit the accessibility of the target protein to the query protein, affecting their interaction. We have developed a new method for assaying protein molecules involving a high-affinity capture tag, the HaloTag (39), to capture a single-stranded DNA oligonucleotide barcode connected to a synthesized protein. We called this system ‘HaloTag barcode assay’ (Figure 1B). This technology enables determination of the number of protein molecules with a wider than heretofore sensitivity range using the protein-attached oligonucleotide. The assay was designed to facilitate proper protein folding, as the capture does not require any solid support, such as polysaccharide polymer beads, glass microscope slides, etc. HaloTag-based protein labeling has several advantages over other labeling approaches, e.g. ones relying on the biotin–avidin interaction, as a useful tool for protein labeling. HaloTag-labeled proteins irreversibly bind to a small chemical ligand chloroalkane in a 1:1 ratio and, hence, the correct numbers of the fused protein molecules can be easily determined. By contrast, a single molecule of avidin can interact non-covalently with up to 4 biotin molecules (40). The size of the HaloTag (33 kDa) is relatively small compared with that of avidin (∼70 kDa) or streptavidin analogs (∼50 kDa), the latter of which could interfere with complex formation during protein interactions. In addition, as the used oligonucleotide also binds the HaloTag in a 1:1 ratio (instead of 1:4, as for of biotin–avidin binding), the small size of the protein–oligonucleotide conjugate by HaloTag compared to biotin-avidin binding allows for appropriate interactions of the labeled protein with other proteins. A previously developed HaloTag-mediated conjugation method used a 220-bp double-stranded DNA with Acrydite modification to label protein (28). We have modified the system by using a newly developed 100mer oligonucleotide containing a unique molecule counting barcode of a semi-random sequence, protein identifier, and a sequencing adaptor (Figure 1A, Supplementary Table S1). To link the HaloTag fusion protein with the barcode, we created amine-modified 100mer oligonucleotides conjugated with HaloTag O4 ester ligand (see Materials and Methods for details). After purification of the ligand–oligonucleotide complex, the HaloTag fusion protein is covalently bound to a ligand–oligonucleotide complex (Figure 1B). We purified the DNA–HaloTag fusion protein complex, the ‘DNA-barcoded protein’ by HPLC, and used it to determine the protein numbers (Figure 1B). Since protein activity depends on its ability to complex with other proteins, the folding of a multidomain protein in any organism is important for its function. To test whether the activity of the HaloTag-fused protein bound with an oligonucleotide DNA-barcoded protein was retained, we used two pairs of well-studied protein interaction pairs, protein G-IgG and JUN-FOS. Using the HaloTag barcode assay, we conjugated DNA barcodes with protein G and FOS (Supplementary Figure S2A and B). We then tested the interaction between these barcoded proteins and their known interacting proteins, IgG and JUN, respectively; this was combined with qPCR quantification. Indeed, both barcoded proteins interacted with their respective protein partners, in agreement with previous reports (Figure 2A and B) (29,41). This demonstrated that functional, full-length target fusion-barcoded proteins are most likely properly folded and capable of interacting with other proteins.

### The sensitivity of the HaloTag barcode assay

To compare the sensitivity of the HaloTag DNA barcode assay and that of conventional luciferase assay involving NanoLuc proteins, we prepared five amine-modified barcode oligonucleotides (Supplementary Table S1, Oligo01, 03, 04, 05 and 06) and created three DNA-barcoded protein constructs (HaloTag, HaloTag-JUN and HaloTag-FOS fusions) conjugated with NanoLuc protein (NL-HaloTag, NL-HaloTag-JUN and NL-HaloTag-FOS, respectively) (42). These complexes were prepared in a dilution series based on qPCR quantification, and complex levels were simultaneously assayed by DNA barcode sequencing and luciferase activity determination (see Materials and Methods and Supplementary Tables S5 and S6). A direct comparison of the two assays for NL-HaloTag is shown in Figure 2C and D. The HaloTag barcode assay was able to detect 1000-fold less protein than the NanoLuc luciferase assay. Similarly, the sensitivity of HaloTag assays with NL-HaloTag-JUN or -FOS fusion proteins was higher (approximately 100-fold) than that of the luciferase assay, although the sensitivity was smaller than that of the NL-HaloTag fusion protein (∼1000-fold) in both assays (Figure 2C and D). This suggests that the large size of a protein or protein fusion can reduce the sensitivity of both assays as the size may inhibit luciferase activity and hinder the amplification of the barcode DNA fused to the proteins. The reads of barcoded proteins that exceeded 10 units (Supplementary Table S5) above the background level were considered to be valid, indicating that the assayed proteins were highly abundant (Figure 2D and Supplementary Tables S5 and S6). The reads were scored as background noise if the mapped reads of barcodes used in the assay were less than 3 SD+average (Supplementary Table S5) of the unmapped barcode reads, which were not used in the assay. The superior sensitivity of the HaloTag assay is most likely associated with the appreciably higher sensitivity of DNA amplification by PCR than that of bioluminescence, which enables the capture of even single molecules of the barcoded protein, with improved efficiency of detection of the barcoded protein. The HaloTag barcode assay highlights the advantages of using DNA instead of the conventional assays to detect protein levels.

### Detecting protein interactions by click-HaloTag barcode assays

**Figure 3.**
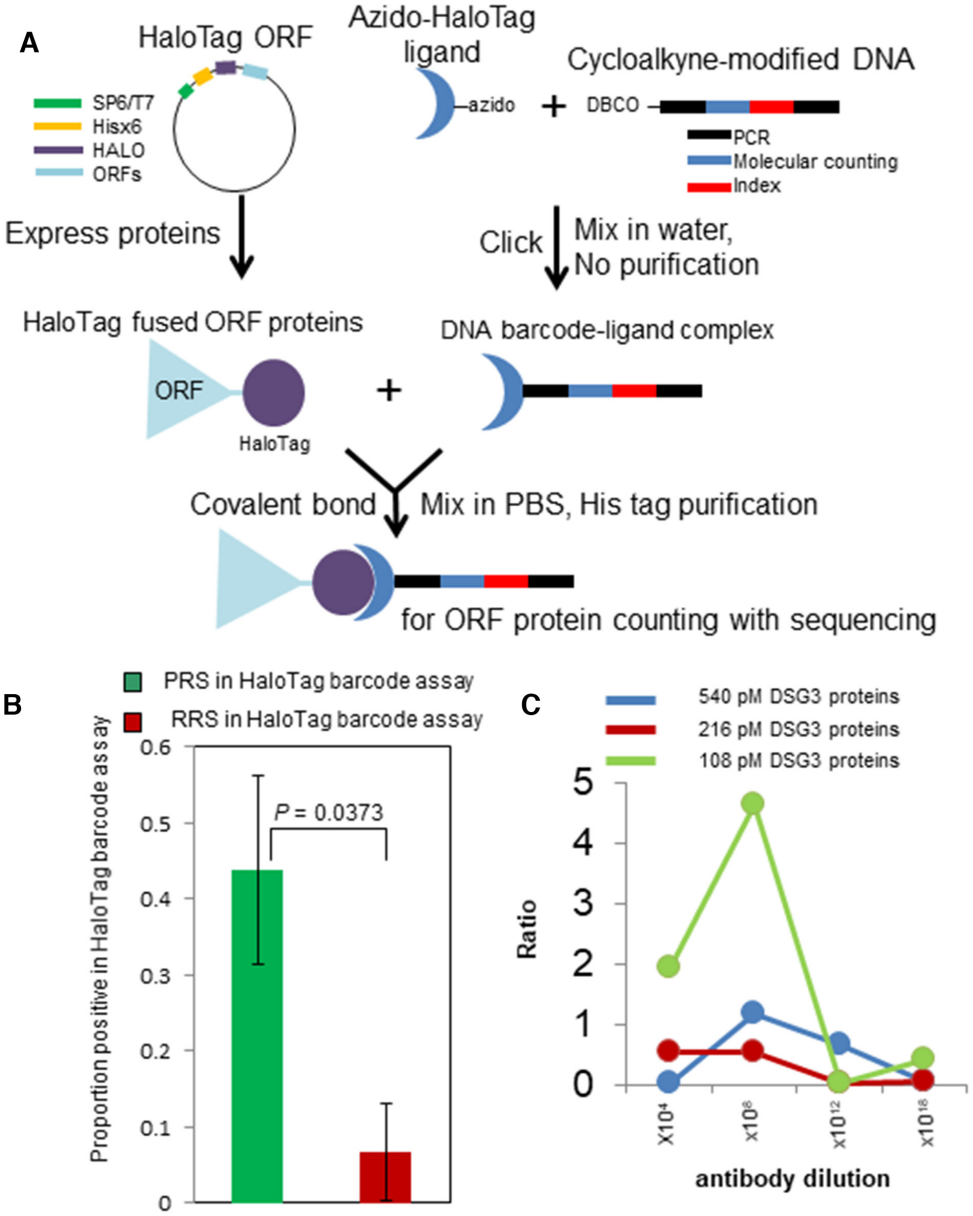
Application of HaloTag-based barcoding. (**A**) Click reaction-based HaloTag protein barcoding. The HaloTag protein covalently interacts with custom azido-chloroalkane ligands conjugated with cycloalkyne-modified DNA barcodes. (**B**) PPI replicated by the HaloTag protein barcoding assay. The proportion of positive scoring pairs within a set of known positive interactions, and a set of randomized sample pairs of interactions for which no evidence of interaction was reported (11). Error bars: SD of the proportion. The number of known positive interacting pairs was significantly higher than that of sample pairs among the randomized pairs (*P* = 0.0373, Fisher's exact test, two-tailed). (**C**) Detection of pemphigus immunoglobulin by using the barcoded antigen DSG3. PRS: positive reference set, i.e. known positive interactions. RRS: random reference set, i.e. randomized pairs of interactions that to date have not been supported by experimental evidence.

Understanding the physical PPI is key for understanding cellular signaling networks, e.g. transcription machinery or proteasome degradation. Therefore, as a first test of the developed HaloTag barcode assay, we focused on PPIs that had been described previously (11,43). Further, for this particular protein profiling method, we developed a click chemistry based conjugation approach for preparing HaloTag-barcoded proteins, the click-HaloTag barcoding (Figure 3A). Compared with the amine-ester reaction described above, the click-HaloTag barcode conjugation method is cost- and time-effective and may be conducted in water with no chromatography purification steps required. For the conjugation, we examined the ability of two robust custom azido-HaloTag ligands (AzHLT-1 and AzHLT-2; Supplementary Figure S1A and Supplementary Methods) (31) to bind with cycloalkyne-modified oligonucleotides. No ligand-oligonucleotide conjugate purification by HPLC was required, and the HaloTag fusion proteins were directly covalently bound to the ligand-oligonucleotide complex. Although both azido-HaloTag ligands could be used for the conjugation of the complex, the AzHLT-1 ligand, with a relatively more reactive alkyl azido group, captured up to five times more protein than the AzHTL-2 ligand with an aromatic azido group, as revealed by direct detection of captured proteins using a tetramethylrhodamine fluorescent (TMR)-modified 100-bp oligonucleotide (Supplementary Figure S1B). After conjugating DNA and protein using the AzHTL-1 ligand, we confirmed that no active HaloTag was present in the reaction mixture although non-barcoded HaloTag proteins were indeed observed (Supplementary Figure S1C, lane 2, and Supplementary Figure S1D, lane 2). The developed click-conjugation method was also used to modify crude proteins, expressed *in vitro* using the wheat germ extract system (Supplementary Figure S3). The results of HaloTag protein staining using TMR ligand and DBCO-modified TMR are shown in Supplementary Figure S3 (lanes A1–A5 and B1–B5). Because the DNA oligonucleotide conjugation occupies the HaloTag ligand pockets, the non-barcoded HaloTag protein staining is undetectable (Supplementary Figure S3, lanes A1–A5 and B1–B5). It revealed that the conjugation method generated a complex with no free binding pocket for the HaloTag ligand and no HaloTag protein linked to an azide ligand only (no DNA) under the experimental conditions tested (1:2:4 mol/mol/mol ratio).

To demonstrate that a click-HaloTag-barcoded protein can be used for protein profiling, the generated protein-oligonucleotide complexes were individually His-tag–purified and used in a PPI assay. To determine the sensitivity and background noise of the click-HaloTag barcode PPI assay, we prepared 80 barcode oligonucleotides (Supplementary Table S1), and evaluated a small set of known positive interactions (PRS) and a set of randomized pairs of interactions that to date have not be supported by experimental evidence (RRS; 11). Of the 16 known positive interactions evaluated by the HaloTag barcode PPI assay, several pairs were scored as positive (44%). This detection rate was appreciably higher than that of the previously reported single pair determined to be positive in a set of 16 randomized pairs of interactions (11) (Supplementary Table S3). The number of known positive interactions that tested positive in the barcode assay was statistically different from that of randomized pairs (Figure 3B, *P* = 0.0373, Fisher's exact test, two-tailed). These observations indicated that the PPI assay using HaloTag-barcoded proteins constructed by click chemistry-based technology is reliable and capable of detecting PPI with a success rate that is only slightly lower than that of a conventional pull-down assay (63%) (11). The significant but relatively lower reproducibility (44%) in the PRS is possibly explained by our previous observation (44) that different interaction assays detect different ‘ true ’ interactions, generally as a result of different assay biochemistry, fusion tags including DNA oligonucleotide barcode, and so on. We tested the high-throughput assay in a prepared 51 barcoded protein mixture. Protein query bZIP53 (AT3G62420) on the Halo magnetic beads heterodimerized related family proteins bZIP63 (AT5G28770), in agreement with previous reports of bZIP53 heterodimerization (45–47) (Supplementary Figure S4, Table S4). These results indicated that a barcoded protein mixture in PBS is likely to be properly folded and capable of specific interactions. Among the 51 barcoded proteins in the mixture, we also detected binding of bZIP53 to other novel proteins, such as AT3G08500 MYB transcription factor and AT5G06950 TGA2 bZIP transcription factor family protein.

### Detection of pemphigus immunoglobulin using DSG3-linked DNA barcode

**Figure 4.**
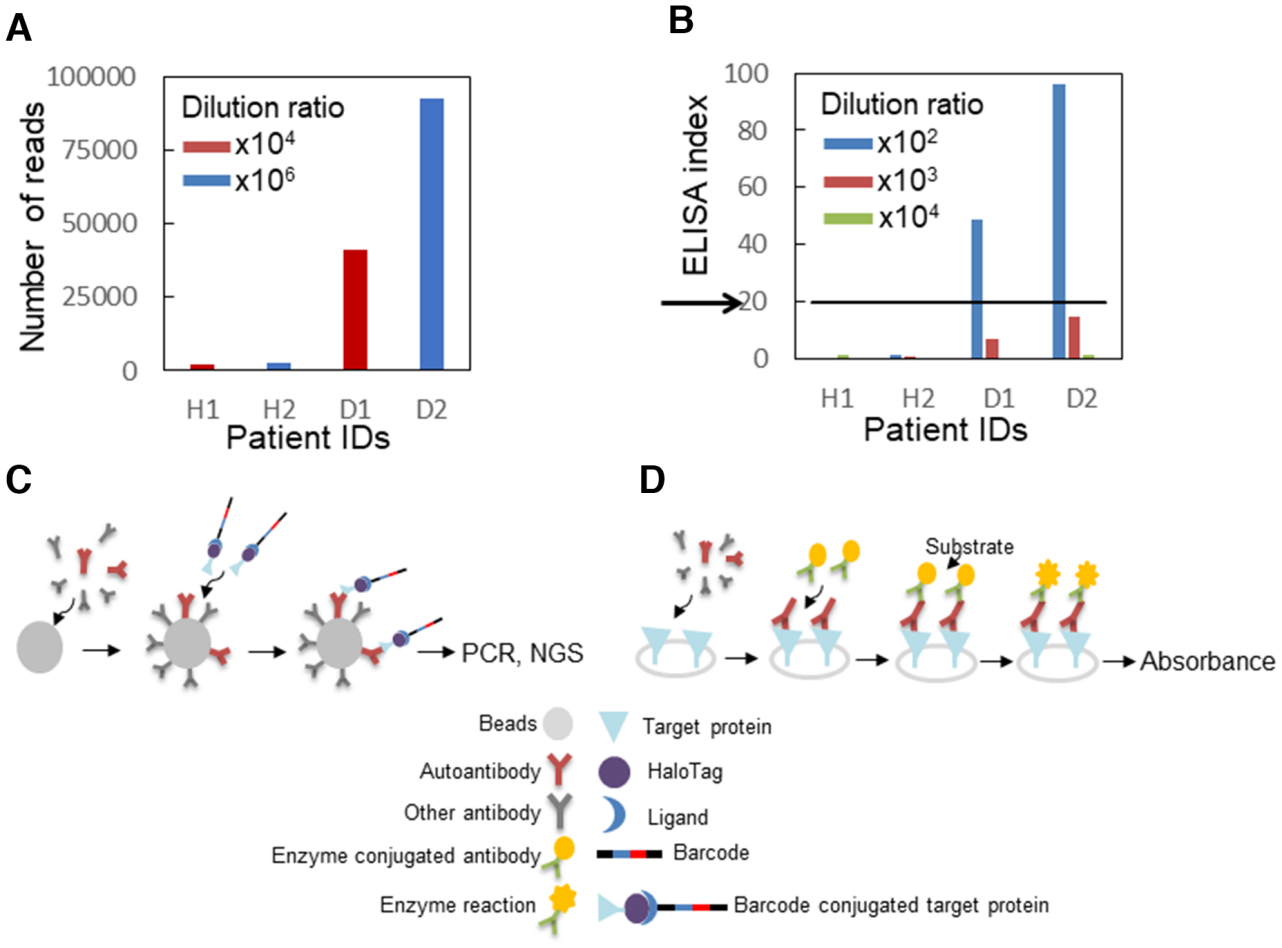
Autoimmune antibody detection (**A**) Detection by barcoding assay. The number of reads (the antigen–antibody interaction signal) is shown on the y-axis. The patient's ID is shown on the x-axis. The serum dilution ratio was 10^4^ for H1 and D1 (red), and 10^6^ for H2 and D2 (blue). (**B**) Detection by conventional ELISA. Quantification of the signal from the ELISA for Desmoglein 3 (DSG3) with the clinical specimens. The ELISA index (the antigen-antibody interaction signal) is shown on the y-axis. The patient's ID and the antibody dilution ratio are shown on the x-axis. The serum dilution ratio was 10^2^ (blue), 10^3^ (red) and 10^4^ (green). The ELISA index from clinical specimens above 20 is indicated positives according to the manufacturer's recommendations (black arrows). Patient ID H1: healthy 1, H2: healthy 2, D1: PV patient 1, D2: PV patient 2. (**C**) A framework for Immunoprecipitation with DNA oligonucleotide-barcoded proteins. The antibody from clinical specimens containing the autoantibody is mixed with antibody capture beads, such as protein G beads. The addition of DNA-conjugated target proteins (antigen) results in an interaction between the autoantibody and the antigen target proteins. DNA conjugated to the target proteins via the HaloTag enables the detection of antibody numbers by quantitative PCR or NGS. (**D**) A framework for conventional ELISA assay. The antibodies from clinical specimens are applied and allowed to interact to anchor antigen proteins on a solid support. The addition of the enzyme-conjugated antibodies (e.g. Horse Radish Peroxidase) and the substrates results in a characteristic color change that can be detected by spectrophotometric methods.

Detection of autoantibodies during an initial stage of any disease is critical for early diagnosis, to avoid invasive inspection. PV is a rare autoimmune disease that causes mucocutaneous blister mediated by autoantibody targeting a cell-cell adhesion molecule (48,49). Detection of a specific autoantibody against the main target autoantigen, DSG3, in the patient's serum by ELISA is a key serological diagnostic criterion for appropriate assessment of the disease. To develop a method for detecting the autoantibody with improved sensitivity, we created barcoded DSG3 protein by using click chemistry. Using a monoclonal anti-DSG3 antibody generated from the PV model mouse as a mimic of the autoantibody from human patients, we compared the dynamic range of the HaloTag barcode assay and that of conventional ELISA. The results of the barcode assay using HaloTag-fused DSG3 are shown in Figure 3C and Supplementary Table S7. The assay showed the greatest sensitivity when a 10^8^ anti-DSG3 antibody dilution was used. The HaloTag barcode method detected concentrations of the antibody that were up to 10^4^ times lower than those detected by conventional ELISA (10^3^ as the highest detected dilution; Supplementary Figure S5A) although DSG3 protein was fused with a 33 kDa HaloTag and 100-bp single-stranded DNA. We considered the possibility that the commercial ELISA assay kit detected the structure of DSG3 protein captured in the assay plate well that was different from that of barcoded DSG3 protein. To address this potential structural artifact, we immobilized the HaloTag fusion DSG3 protein on a HaloTag ligand-coated 96 well plate (the HaloLink plate). The HaloLink plate (Promega) captures approximately 10 ng/mL of HaloTag-GST (50). Similar to the ELISA plate, the maximum signal/noise range of 1 ng of HaloTag fusion DSG3 was observed for 10^4^ antibody dilution (Supplementary Figure S5B). Although we were able to detect protein-protein interactions including antigen–antibody interactions, whether the assay would improve real-life diagnosis needed further investigation. Therefore, we applied the barcode assay to determine the presence of the anti-DSG3 antibody in two patients with PV and two healthy volunteers. Our detection system showed superior sensitivity and could detect the presence of the anti-DSG3 antibody in the patient serum, which was diluted 10^6^ times Figure 4A and Supplementary Table S8), while conventional ELISA had a lower detection limit at a dilution of 10^2^ times (Figure 4B). The sensitivity of the barcode assay could be attributed not only to the high sensitivity of DNA amplification but also to antibody enrichment by bead capture in aqueous solution, as compared with dispersed antigen-capture on the ELISA plate. In addition, in the ELISA plate assay, the target protein is anchored to a solid support, which may limit the accessibility of the antibody to the target protein, whereas the barcode assay does not involve such anchoring. Hence, the barcoded antigen can capture more antibodies, resulting in a more efficient interaction than ELISA. These observations suggest that the developed method could be used for an extremely early-stage diagnosis of pemphigus based on the detection of autoantibodies in clinically-suspected pemphigus patients.

## DISCUSSION

In the current study, we developed an alternative DNA-protein conjugation methodology, click-based HaloTag protein barcoding, and used it to detect PPIs, including antibody-antigen interactions. Another protein labeling technique that is commercially available is a SNAP-tag (19 kDa), which covalently links with a suitable ligand, benzylguanine derivatives (51). This ligand can be modified with various functional groups such as fluorophores or beads. It might have been useful to link proteins with a single-stranded oligonucleotide instead of HaloTag. However, because large HaloTag ORF collections already exist for two organisms, human (52) (http://www.kazusa.or.jp/kop/halotag/) and Arabidopsis (10) (https://www.arabidopsis.org/abrc/halo_tagged_orf_clones.jsp), all assays in the current study involved HaloTag barcoding. Using a protein interaction assay involving known positive protein pairs, we demonstrated that the developed conjugation assay detects significantly more valid interactions than negative interactions. This suggested that PPIs are likely not affected by the conjugation of 100mer oligonucleotides to proteins. However, we were unable to exclude the possibility that the target (prey) or bait protein itself could bind to a random conjugated DNA barcode. Therefore, interaction studies using the developed methods should be performed with suitable caution and should be followed by additional validation. Regardless of the possible nonspecific DNA conjugation to proteins, the main reason for performing the assays in aqueous solutions and with improved sensitivity is the detection of low-copy number proteins. The range of molecule numbers that can be counted by using HaloTag barcoding (Supplementary Table S5, limit of 3.3 molecules from background average) are most likely the same as those of other technologies, such as zero-mode waveguide, total internal reflection microscopy, and single molecule ELISA, which enable the detection of a single fluorescent molecule (53,54). Although the reflection microscopy enables observation of a single molecule in real-time, an important reason for using the barcoding assay is the ability to efficiently quantify protein molecules without fluorescent diffusion. The indispensable NGS technology is widely available and eliminates the need to prepare a solid support for micro/nanofluidic technology. Hence, introduction of bias may be avoided by using the barcoding assay.

The lower sensitivity of detecting HaloTag fusion proteins compared to that of HaloTag alone suggests that the size and/or structure of protein could affect the sensitivity of the barcoding assay. Hence, the small size of a single-stranded oligonucleotide barcode potentially facilitates the interaction with a target antibody or protein, by avoiding access constraints to the target protein that may be imposed by double-stranded DNA. Considering that the diameter of the HaloTag protein is 3.3 nm, using a 100-bp oligonucleotide, a flexible 1-nm persistence length, would result in a relatively small size of a barcoded protein complex in comparison with the same-length double-stranded DNA (33 nm), a helix structure of 50-nm persistence length (150 bp) (55,56). However, we cannot completely exclude the possibility that the relatively low rate (44%) of positive PPI detection by the barcode assay in comparison with the previously reported detection rate of a pull-down assay (63%) (11), might be associated with the barcode. Such lower rate might be caused not only by structural limitations but also by the negatively charged oligonucleotide, which could change some target protein characteristics, such as isoelectric point or hydrophobicity. The HaloTag barcoding technology would be readily available to detect PPI in a multiplex assay with multiple barcoded proteins. For example, multiplexed barcoded antigen proteins DSG1 and 3, which are functionally compensatory, could be utilized for a quantitative and sensitive detection to distinguish two autoantibodies related to PV and pemphigus foliaceus (57). The main advantages of the assay are the aqueous solution-based multiplexing detection capability and superior sensitivity. Since the system uses an aqueous-based assay, there is no limitation for the number of multiplexed barcoded proteins that can be used. The aqueous system does not limit the access to the antibody, and the barcoded antigens bind to the antibodies in the test sample with very high sensitivity. The click-HaloTag barcode conjugation eliminates the cumbersome steps of conjugation complex formation, pH adjustments, purification by chromatography, and the use of organic solvents. However, the click chemistry based conjugation methods still require the expression of each prey protein separately prior to the assay. Also, all complexes formed should be purified separately similar to that in the amido bond-based method.

Although the HaloTag system has a smaller conjugation size than that of the biotin/avidin system, it is not ‘small.’ A 33 kDa HaloTag is larger than a GFP tag and may interfere with the protein–protein interaction as well. However, the 1:1 conjugation ratio of the HaloTag prevents improper folding of barcoded proteins because of the small complex size, as compared with other conjugation methods, e.g. the widely used biotin–avidin (1:4) interaction and non-scalable amine–ester protein (antibody) barcoding (13,15). Further, the developed click chemistry-based HaloTag DNA linking methods may help improve protein capture on the HaloTag protein array (11) and be used instead of the bissulfosuccinimidyl suberate crosslinker, which forms a paste during spotting solution preparation (10). Using the linking methods, DNA-conjugated HaloTag-fused protein could be captured on an amine-coated solid support by the ligand–oligonucleotide complex instead of bissulfosuccinimidyl suberate. Using this method to evaluate a set of barcoded proteins to identify their target proteins, such antigen/antibody pairs could constitute an accurate detection approach, with superior sensitivity and throughput. We demonstrate that the applicability of the HaloTag barcoding assay and the technique add a new dimension to protein detection for an especially small number of target proteins, such as autoantibodies, because of the higher assay sensitivity due to the use of nucleic acids, compared to conventional assays that use fluorescent molecules or enzyme reactions (Figure 4C, D). For the clinical diagnosis of real-life conditions such as PV in the future, the false-discovery rate of the assay should be estimated, and the sensitivity should be determined by benchmarking against conventional chemiluminescent enzyme immunoassays using sample pairs consisting of patients with PV and healthy volunteers. In conclusion, the developed method can be used to facilitate extremely early-stage diagnosis of human diseases. In addition to barcoding HaloTag fusion proteins, click chemistry-based barcoding can be readily applied to antibodies, of which large collections already exist. We envision that the click-based barcoding technology will be adapted for other applications, including antibody labeling to help identify cell specificity and cancer specificity and contribute to the diagnosis of human disease.

## IN-HOUSE MATERIALS, PROGRAMS AND DATA AVAILABILITY

The two types of azido-HaloTag ligand, AzHLT-1 (31) and AzHLT-2 (Supplementary Method andSupplementary Figure S1A), used to develop the new method are available upon request from T.H. (thosoya.cb@tmd.ac.jp). The scripts of in-house clustering software ‘Nucleotide Sequence Clusterizer’ used for barcode counting are available upon request from K.S. (katsuyuki.shiroguchi@riken.jp). Sequence data generated in the current study were deposited in GEO under the accession number GSE122542 (https://www.ncbi.nlm.nih.gov/geo/query/acc.cgi?acc=GSE122542).

## Supplementary Material

gkz1086_Supplemental_Files
