# Supplementary material for: HaloTag-based conjugation of proteins to barcoding-oligonucleotides
Source: Nucleic Acids Res. 2019 Nov 22;48(2):e8. doi: 10.1093/nar/gkz1086 (PMC6954424; doi:10.1093/nar/gkz1086)
Supplement: gkz1086_Supplemental_Files [file gkz1086_supplemental_files.zip › 03-1_Supplementarydata_revised_part1.pdf]

SUPPLEMENTARY DATA

HaloTag-based conjugation of proteins to barcoding oligonucleotides

Junshi Yazaki[1*], Yusuke Kawashima[1], Taisaku Ogawa[2], Atsuo Kobayashi[1], Mayu Okoshi[1], Takashi Watanabe[1], Suguru Yoshida[3], Isao Kii[4], Shohei Egami[5,6], Masayuki Amagai[5,6], Takamitsu Hosoya[3,7], Katsuyuki Shiroguchi[2,8,9] and Osamu Ohara[1]

[1] Laboratory for Integrative Genomics, RIKEN, Yokohama City, 230-0045, Japan, [2] Laboratory for Prediction of Cell Systems Dynamics, RIKEN Center for Biosystems Dynamics Research (BDR), Osaka, 565-0874, Japan, [3] Laboratory of Chemical Bioscience, Institute of Biomaterials and Bioengineering, Tokyo Medical and Dental University, Tokyo, 101-0062, Japan, [4] Common Facilities Unit, Compass to Healthy Life Research Complex Program, RIKEN Cluster for Science, Technology and Innovation Hub, Kobe, 650-0047, Japan, [5] Laboratory for Skin Homeostasis, RIKEN Center for Integrative Medical Sciences (IMS), Yokohama 230-0045, Japan, [6] Department of Dermatology, Keio University School of Medicine, Tokyo, 160-8582, Japan, [7] Laboratory for Chemical Biology, RIKEN Center for Biosystems Dynamics Research (BDR), Kobe 650-0047, Japan, [8]Laboratory for Immunogenetics, RIKEN Center for Integrative Medical Sciences (IMS), Yokohama 230-0045, Japan, [9] JST PRESTO, Kawaguchi 332-0012, Japan.

* To whom correspondence should be addressed. Tel: +81 452-503-9696; Fax: +81 452-503-9694; Email: junshi.yazaki@riken.jp

SUPPLEMENTARY METHODS

Chemical synthesis of

4-Azido-*N*-(2-(2-(2-(2-(4-(4-((2-(2-((6-chlorohexyl)oxy)ethoxy)ethyl)carbamoyl)phenyl)-1*H*-1,2,3-triazol-1-yl)ethoxy)ethoxy)ethoxy)ethyl)benzamide (AzHTL-2)

General notes: The reaction was performed in dry glassware under atmosphere of argon. Analytical thin-layer chromatography (TLC) was performed on pre-coated (0.25 mm) silica-gel plates (Merck Chemicals, USA, silica gel 60 F_{254}, cat. no. 1.05715). Preparative TLC (PTLC) was performed on silica-gel (Wako Pure Chemical Industries Ltd. Japan, Wakogel B5-F, cat. no. 230-0043). Melting point (Mp) was determined by using an OptiMelt MPA100 automated melting point apparatus (Stanford Research Systems, USA) and is uncorrected. IR spectrum was acquired by diffuse reflectance method on a Shimadzu IRPrestige-21 spectrometer attached to DRS-8000A (Shimadzu, Japan) with the absorption band given in cm^{-1}. ^{1}H and ^{13}C nuclear magnetic resonance (NMR) spectra were acquired using a Bruker AVANCE 500 spectrometer at 500 and 126 MHz, respectively. Further, $CDCl_3$ (99.8%D, Kanto Chemical Co. Inc. Japan, cat. no. 07663-23) was used as a solvent for obtaining NMR spectra. Chemical shifts (δ) are given in parts per million (ppm) downfield from $(CH_3)_4Si$ (δ 0.00 for ^{1}H NMR in $CDCl_3$) as an internal reference with coupling constants (*J*) in hertz (Hz). The abbreviations s, d, t, m, and br signify singlet, doublet, triplet, multiplet, and broad, respectively. High-resolution mass spectra (HRMS) were acquired using a microTOF mass spectrometer (Bruker, Germany) under positive electrospray ionization (ESI^+) conditions.

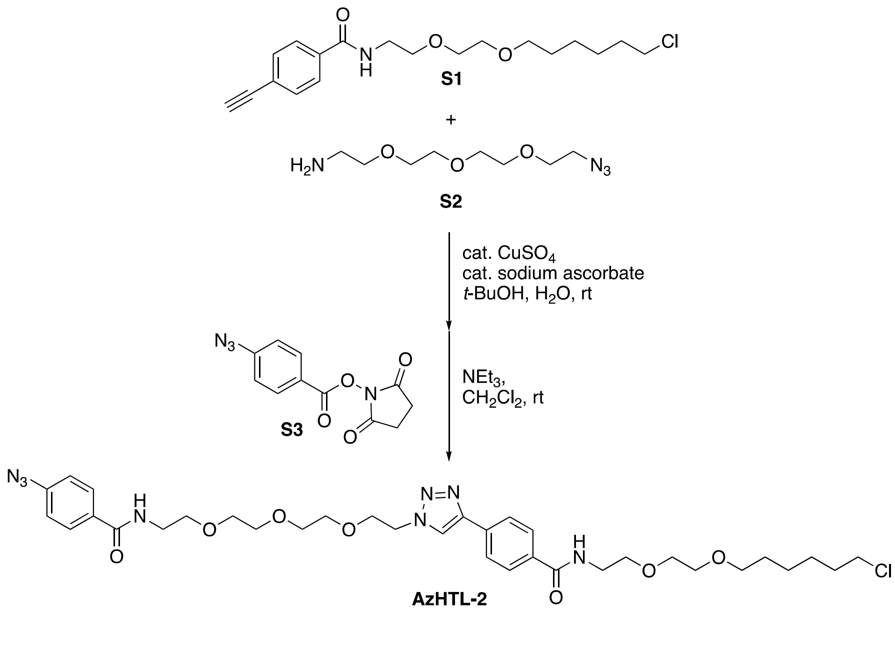

Synthesis: 11-Azido-3,6,9-trioxaundecan-1-amine (**S1**) and *N*-(2-(2-((6-chlorohexyl)oxy)ethoxy)ethyl)-4- ethynylbenzamide (**S2**) were prepared as previously reported (1). To a mixture of **S1** (20.4 mg, 93.6 μmol) and **S2** (33.0 mg, 93.8 μmol) in *t*-BuOH (0.50 mL) and water (0.50 mL) were added $CuSO_4 \cdot 5H_2O$ (1.3 mg, 5.2 μmol) and sodium ascorbate (2.0 mg, 10 μmol) at room temperature (RT, 25°C). After stirring for 24 h at the same temperature, the mixture was concentrated under reduced pressure. To the mixture were added CH_2Cl_2 (1.0 mL), 2,5-dioxopyrrolidin-1-yl 4-azidobenzoate (**S3**) (13.0 mg, 50.0 μmol), and triethylamine (7.0 μL, 51 μmol) at RT. After stirring for 6 h at the same temperature, the mixture was concentrated under reduced pressure. The residue was purified by PTLC (CH_2Cl_2/MeOH = 10/1) to give **AzHTL-2** (18.8 mg, 26.3 μmol, 28.1%) as a pale yellow solid. Mp 74–76°C; TLC R_f 0.53 (CH_2Cl_2/MeOH = 10/1); ^1H NMR ($CDCl_3$, 500 MHz) δ 1.30–1.44 (m, 4H), 1.58 (tt, 2H, *J* = 7.5, 7.5 Hz), 1.72 (tt, 2H, *J* = 7.5, 7.5 Hz), 3.46 (t, 2H, *J* = 6.5 Hz), 3.49 (t, 2H, *J* = 6.5 Hz), 3.54–3.75 (m, 22H), 4.57 (t, 2H, *J* = 5.0 Hz), 6.80 (br s, 1H), 6.84 (br s, 1H), 7.04 (d, 2H, *J* = 8.5 Hz), 7.80 (d, 2H, *J* = 8.5 Hz), 7.85 (d, 2H, *J* = 8.0 Hz), 7.89 (d, 2H, *J* = 8.0 Hz), 8.04 (s, 1H); ^{13}C NMR ($CDCl_3$, 126 MHz) δ 25.4 (1C), 26.7 (1C), 29.4 (1C), 32.5 (1C), 39.7 (1C+1C, two signals overlapped), 45.0 (1C), 50.4 (1C), 69.4 (1C), 69.7 (1C), 70.0 (1C+1C, two signals overlapped), 70.1 (1C), 70.3 (1C), 70.4 (1C), 70.45 (1C), 70.51 (1C), 71.3 (1C), 118.9 (2C), 121.6 (1C), 125.6 (2C), 127.6 (2C), 128.9 (2C), 131.0 (1C), 133.7 (1C), 133.9 (1C), 143.2 (1C), 146.7 (1C), 166.4 (1C), 167.0 (1C); IR (KBr, cm^{-1}) 851, 1047, 1117, 1285, 1352, 1458, 1499, 1539, 1643, 2124, 2864, 2934, 3329; HRMS (ESI$^+$) *m*/*z* 737.3126 ([M+Na]$^+$, $C_{34}H_{47}{}^{35}ClN_8NaO_7{}^+$ requires 737.3148).

SUPPLEMENTARY FIGURE LEGENDS

Supplementary Figure S1. Representative click-based protein conjugation.

Supplementary Figure S2. Representative protein conjugation with DNA.

Supplementary Figure S3. Application of the conjugation methods to process crude proteins expressed *in vitro* using the wheat germ extract system.

Supplementary Figure S4. Reads count from a protein-protein interaction (PPI) assay with barcoded protein mixture is shown.

Supplementary Figure S5. Applications of HaloTag-proteins.

SUPPLEMENTARY TABLES

Supplementary Table S1. DNA oligonucleotide sequences for protein indexing, and the counting barcode for the amido-bond–based method and click chemistry-based HaloTag-barcoding.

Supplementary Table S2. Primer sequences for protein indexing and counting library preparation. Index sequences are underlined.

Supplementary Table S3. Summary of the barcode pull-down assays for PPI from literature-curated interactions (Figure 3B).

Supplementary Table S4. Summary of a high-throughput assay with a prepared 51 barcoded protein mixture shown in Supplementary Figure 4.

Supplementary Table S5. Summary of the dynamic range of protein assays by DNA barcode.

Supplementary Table S6. Summary of the dynamic range of HaloTag barcode assay shown in Figure 2D.

Supplementary Table S7. Summary of the DSG3 barcode immunoprecipitation experiment shown in Figure 3C.

Supplementary Table S8. Summary of the PV patient's autoimmune antibody detection with barcoded Desmoglein 3 (DSG3) in Figure 4A.

SUPPLEMENTARY REFERENCES

1. Yoshida,S., Kanno,K., Kii,I., Misawa,Y., Hagiwara,M. and Hosoya,T. (2018) Convergent synthesis of trifunctional molecules by three sequential azido-type-selective cycloadditions. *Chem. Commun.*, **54**, 3705–3708.

2. Yazaki,J., Galli,M., Kim,A.Y., Nito,K., Aleman,F., Chang,K.N., Carvunis,A.R., Quan,R., Nguyen,H., Song,L. et al. (2016) Mapping transcription factor interactome networks using HaloTag protein arrays. *Proc. Natl. Acad. Sci. U S A*, **113**, E4238–4247.

Supplemental figure S1

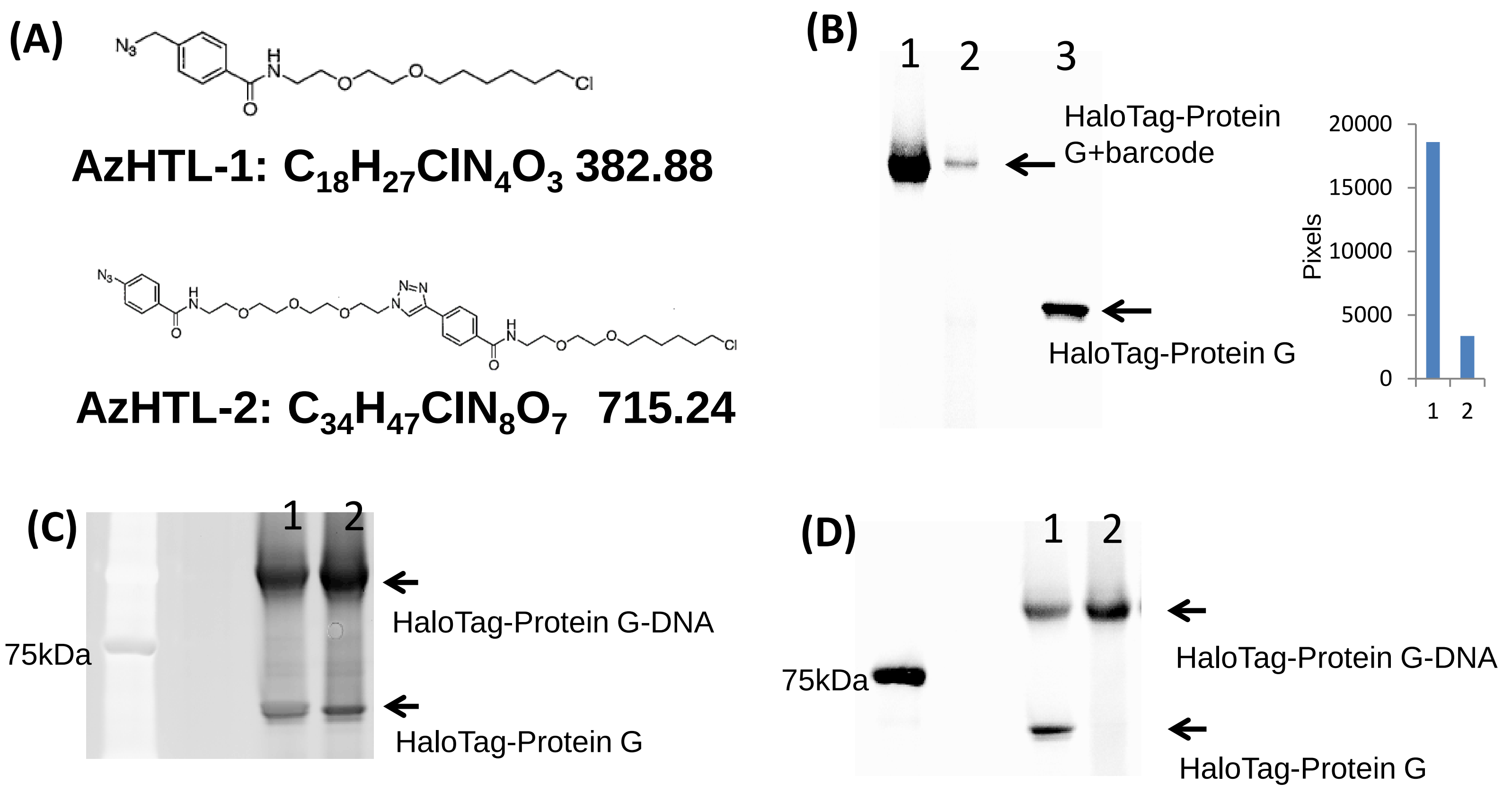

Supplementary Figure S1. Representative click-based protein conjugation. (A) Structure of the azido-HaloTag ligands AzHTL-1 and AzHTL-2. (B) DNA-conjugated protein G using the AzHTL-1 ligand (lane 1) or AzHTL-2 ligand (lane 2), and HaloTag-protein G (lane 3). Protein G was mixed with the DNA barcode and azido-ligand in a 1:1:2 ratio (mol/mol/mol; lanes 1 and 2) and incubated at RT for 1 h. The surface areas of bands corresponding to the gel images on the left are shown in the histogram. (C) HaloTag-protein G conjugated with DNA by click chemistry (lanes 1 and 2) was detected using Sypro Ruby staining. Protein G was mixed with DNA barcode and the azido-ligand AzHTL-1 in a 1:1:2 ratio (mol/mol/mol; lane 1) or 1:2:4 ratio (mol/mol/mol; lane 2), and incubated at RT for 1 h. (D) HaloTag-protein G conjugated with DNA was detected using tetramethylrhodamine (TMR) staining (Promega) and DNA oligonucleotide barcode conjugated with TMR in the gel shown in (C); 75 kDa indicates a molecular mass marker.

Supplemental figure S2

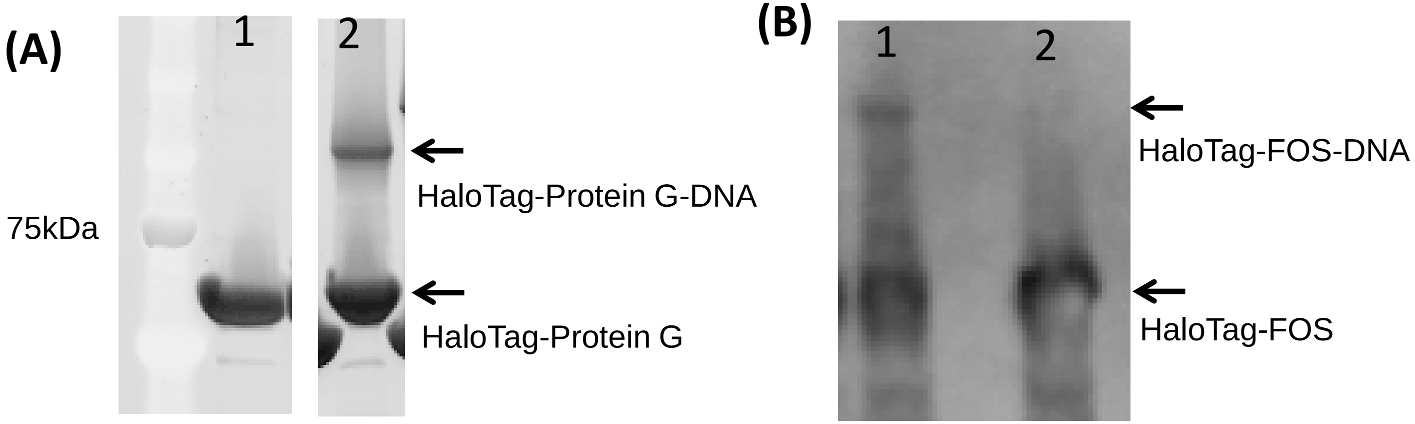

Supplementary Figure S2. Representative protein conjugation with DNA. (A) HaloTagged protein G (lane 1) and protein G conjugated with DNA via the amine-ester reaction (lane 2) were detected using Sypro Ruby staining. Protein G was mixed with the DNA-ligand complex in a 1:2 ratio (mol/mol) and incubated at room temperature (RT, 25° C) for 1 h. (B) HaloTag-FOS conjugated with DNA via the amine-ester reaction (lane 1) and HaloTag-FOS (lane 2) were detected using western blotting with an anti-HaloTag antibody. FOS protein was mixed with the DNA-ligand complex and incubated at RT for 1 h; 75 kDa indicates a molecular mass marker.

Supplemental figure S3

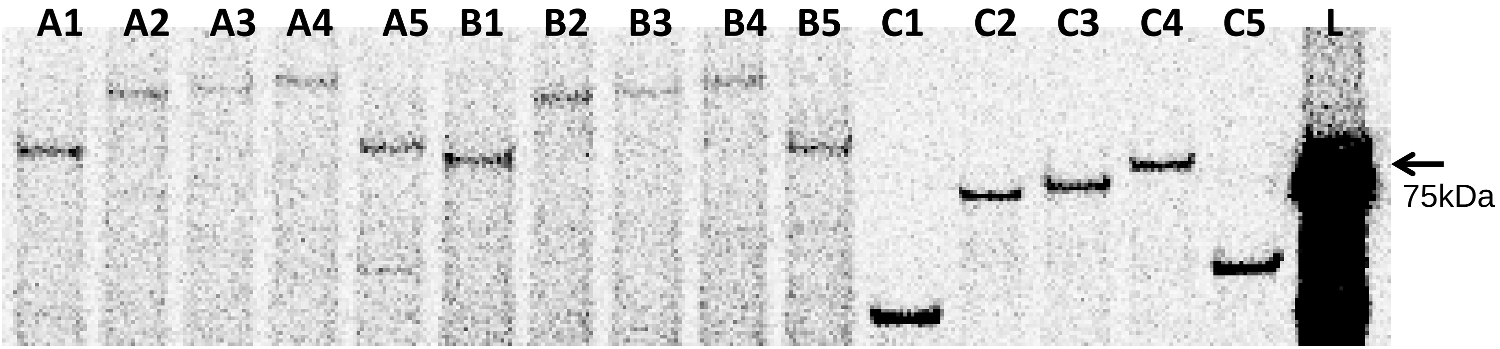

Supplementary Figure S3. Application of the conjugation methods to process crude proteins expressed *in vitro* using the wheat germ extract system. A1–A5 (lanes 1–5), HaloTag-protein conjugated with DNA by click chemistry was detected using TMR ligand staining and DNA oligonucleotide barcode conjugated with TMR. B1–B5 (lanes 6–10), HaloTag-protein conjugated with DNA by click reaction was detected using dibenzocyclooctyne-TMR staining and DNA oligonucleotide barcode conjugated with TMR. C1–C5 (lanes 11–15), HaloTag-protein was detected using TMR ligand staining. L, molecular ladder. All proteins were mixed with a DNA barcode and the azido-ligand AzHLT-1 at a 1:1:2 ratio (mol/mol/mol; lanes A1–B5) and incubated at RT for 1 h. Proteins: 1, At3g62420 (17 kDa); 2, At5g28770 (34 kDa); 3, At5g62380 (40 kDa); 4, At1g12260 (46 kDa); 5, At2G40330 (24 kDa). Protein sizes are specified without the HaloTag (33 kDa). Protein identification numbers correspond to previously assigned *Arabidopsis thaliana* gene identification numbers (2) (https://www.arabidopsis.org/).

Supplemental figure S4

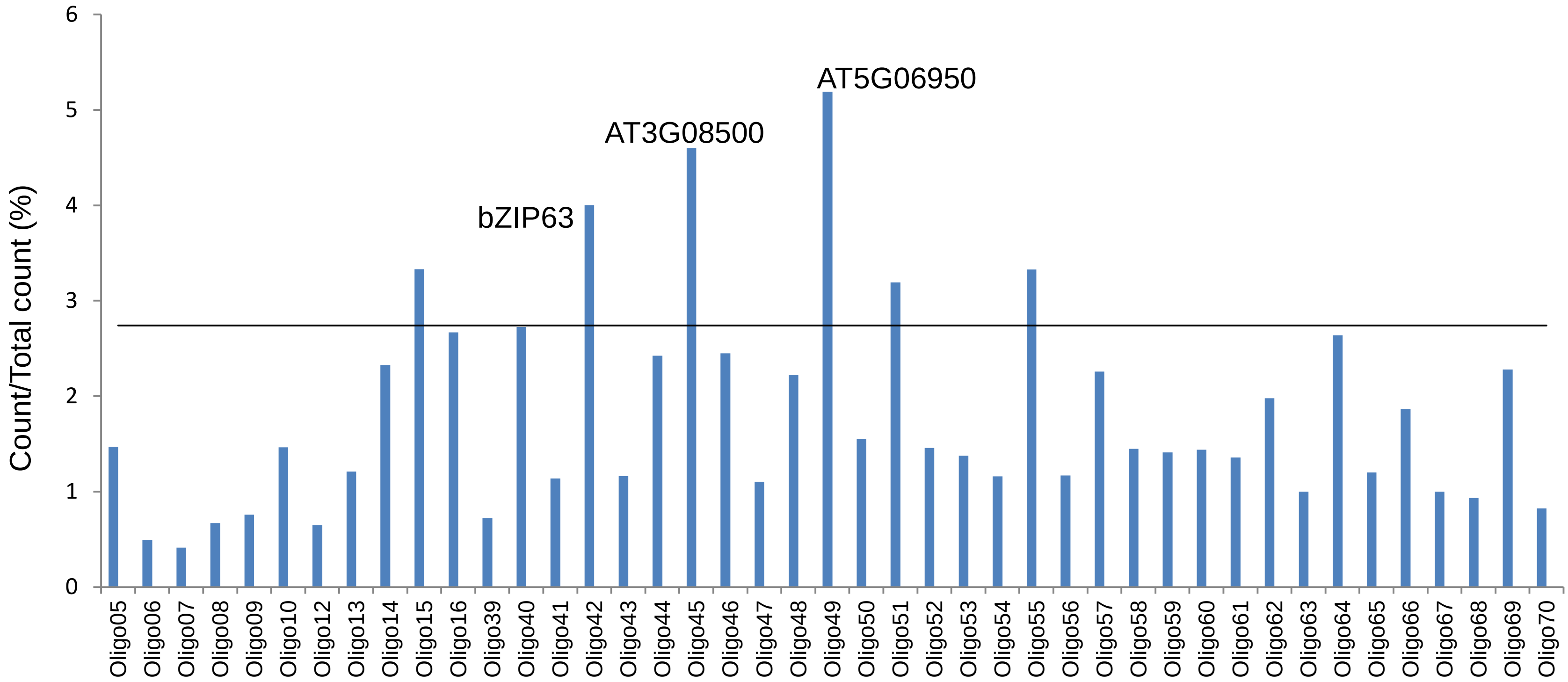

Supplementary Figure S4. Reads count from a protein-protein interaction (PPI) assay with barcoded protein mixture is shown. The histogram indicates read count percentage in a total read count corresponding to data in Supplementary Table S4. The signals from candidate interactors of bZIP53 exceed the average counts of HaloTag-only negative controls (black lines).

Supplemental figure S5

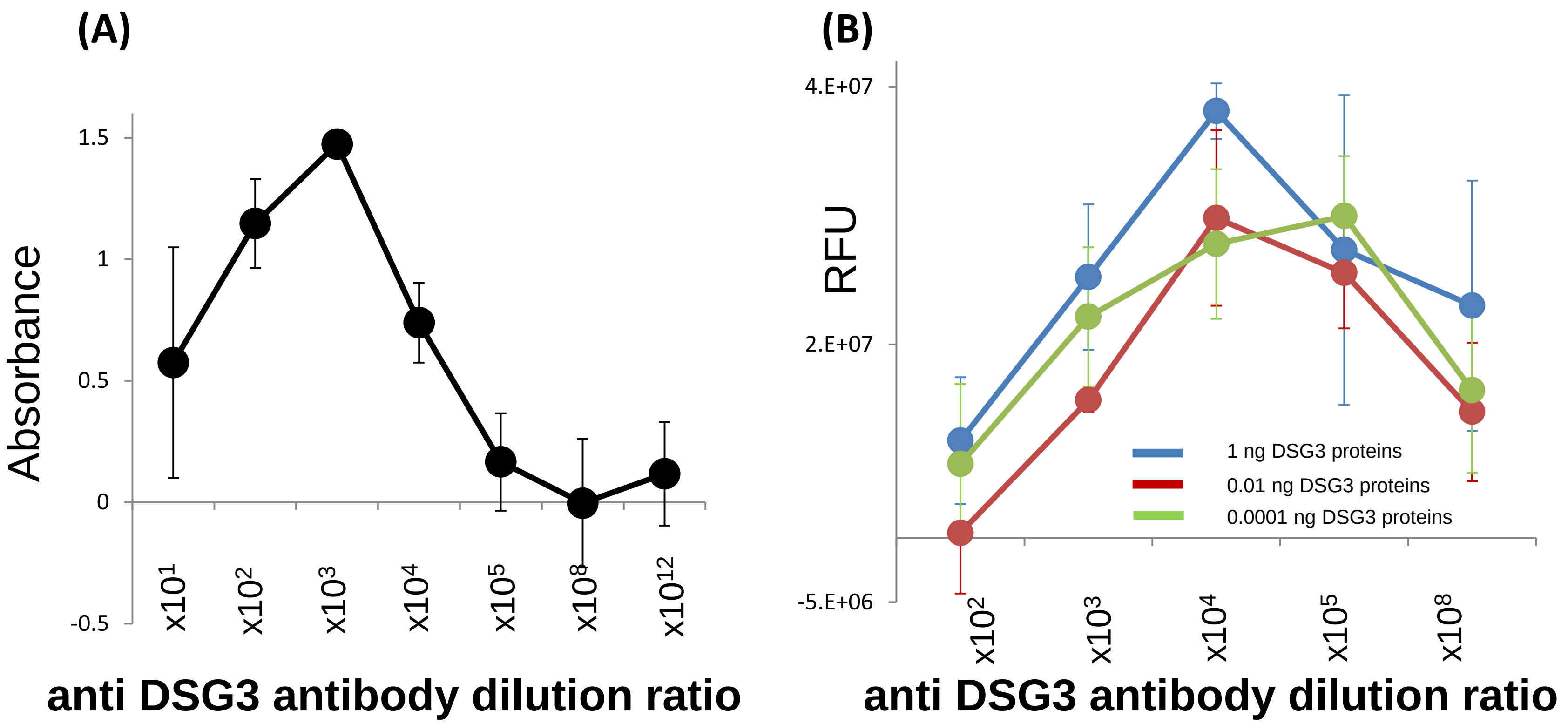

Supplementary Figure S5. Applications of HaloTag-proteins. (A) Quantification of the signal from the enzyme-linked immunosorbent assay for desmoglein 3 (DSG3). The antigen-antibody interaction signal (absorbance at 450 nm) is shown on the y-axis. The anti-DSG3 antibody dilution ratio is shown on the x-axis. Error bars represent the standard error of signal intensity from triplicate experiments. (B) Quantification of the signal from the HaloTag ligand plate assay for DSG3. Relative fluorescence units (RFU) for the antigen-antibody interaction signal are shown on the y-axis. The anti-DSG3 antibody dilution ratio is shown on the x-axis. Error bars represent the standard error of signal intensity.
