# Supplementary material for: HaloTag-based conjugation of proteins to barcoding-oligonucleotides
Source: Nucleic Acids Res. 2019 Nov 22;48(2):e8. doi: 10.1093/nar/gkz1086 (PMC6954424; doi:10.1093/nar/gkz1086)
Supplement: gkz1086_Supplemental_Files [file gkz1086_supplemental_files.zip › 04_SupplementaryTables_part2-2-2.pdf]

Name	PCR primer1 (31mer)	Counting barcode (30mer)	Protein barcode (8mer)	PCR primer2 (31mer)
Oligo01	ACTCTTTCCCTACACGACGCTCTTCCGATCT	NNNNNNN A NN C NNNN T NNNN G NN A NNN C NN	ATTACTCG	AGATCGGAAGAGCACACGTCTGAACTCCAGT
Oligo02	ACTCTTTCCCTACACGACGCTCTTCCGATCT	NNNNNNN A NN C NNNN T NNNN G NN A NNN C NN	TCCGGAGA	AGATCGGAAGAGCACACGTCTGAACTCCAGT
Oligo03	ACTCTTTCCCTACACGACGCTCTTCCGATCT	NNNNNNN A NN C NNNN T NNNN G NN A NNN C NN	CGCTCATT	AGATCGGAAGAGCACACGTCTGAACTCCAGT
Oligo04	ACTCTTTCCCTACACGACGCTCTTCCGATCT	NNNNNNN T NN G NNNN A NNNN C NN T NNN G NN	GAGATTCC	AGATCGGAAGAGCACACGTCTGAACTCCAGT
Oligo05	ACTCTTTCCCTACACGACGCTCTTCCGATCT	NNNNNNN G NN T NNNN C NNNN A NN G NNN A NN	ATTCAGAA	AGATCGGAAGAGCACACGTCTGAACTCCAGT
Oligo06	ACTCTTTCCCTACACGACGCTCTTCCGATCT	NNNNNNN C NN A NNNN G NNNN T NN C NNN T NN	GAATTCGT	AGATCGGAAGAGCACACGTCTGAACTCCAGT
Oligo07	ACTCTTTCCCTACACGACGCTCTTCCGATCT	NNNNNNN A NN C NNNN T NNNN G NN A NNN C NN	CTGAAGCT	AGATCGGAAGAGCACACGTCTGAACTCCAGT
Oligo08	ACTCTTTCCCTACACGACGCTCTTCCGATCT	NNNNNNN A NN C NNNN T NNNN G NN A NNN C NN	TAATGCGC	AGATCGGAAGAGCACACGTCTGAACTCCAGT
Oligo09	ACTCTTTCCCTACACGACGCTCTTCCGATCT	NNNNNNN A NN C NNNN T NNNN G NN A NNN C NN	CGGCTATG	AGATCGGAAGAGCACACGTCTGAACTCCAGT
Oligo10	ACTCTTTCCCTACACGACGCTCTTCCGATCT	NNNNNNN A NN C NNNN T NNNN G NN A NNN C NN	TCCGCGAA	AGATCGGAAGAGCACACGTCTGAACTCCAGT
Oligo11	ACTCTTTCCCTACACGACGCTCTTCCGATCT	NNNNNNN A NN C NNNN T NNNN G NN A NNN C NN	TCTCGCGC	AGATCGGAAGAGCACACGTCTGAACTCCAGT
Oligo12	ACTCTTTCCCTACACGACGCTCTTCCGATCT	NNNNNNN A NN C NNNN T NNNN G NN A NNN C NN	AGCGATAG	AGATCGGAAGAGCACACGTCTGAACTCCAGT
Oligo13	ACTCTTTCCCTACACGACGCTCTTCCGATCT	NNNNNNN A NN C NNNN T NNNN G NN A NNN C NN	TATAGCCT	AGATCGGAAGAGCACACGTCTGAACTCCAGT
Oligo14	ACTCTTTCCCTACACGACGCTCTTCCGATCT	NNNNNNN A NN C NNNN T NNNN G NN A NNN C NN	ATAGAGGC	AGATCGGAAGAGCACACGTCTGAACTCCAGT
Oligo15	ACTCTTTCCCTACACGACGCTCTTCCGATCT	NNNNNNN A NN C NNNN T NNNN G NN A NNN C NN	CCTATCCT	AGATCGGAAGAGCACACGTCTGAACTCCAGT
Oligo16	ACTCTTTCCCTACACGACGCTCTTCCGATCT	NNNNNNN A NN C NNNN T NNNN G NN A NNN C NN	GGCTCTGA	AGATCGGAAGAGCACACGTCTGAACTCCAGT
Oligo17	ACTCTTTCCCTACACGACGCTCTTCCGATCT	NNNNNNN A NN C NNNN T NNNN G NN A NNN C NN	AGGCGAAG	AGATCGGAAGAGCACACGTCTGAACTCCAGT
Oligo18	ACTCTTTCCCTACACGACGCTCTTCCGATCT	NNNNNNN A NN C NNNN T NNNN G NN A NNN C NN	TAATCTTA	AGATCGGAAGAGCACACGTCTGAACTCCAGT
Oligo19	ACTCTTTCCCTACACGACGCTCTTCCGATCT	NNNNNNN A NN C NNNN T NNNN G NN A NNN C NN	CAGGACGT	AGATCGGAAGAGCACACGTCTGAACTCCAGT
Oligo20	ACTCTTTCCCTACACGACGCTCTTCCGATCT	NNNNNNN A NN C NNNN T NNNN G NN A NNN C NN	GTACTGAC	AGATCGGAAGAGCACACGTCTGAACTCCAGT
Oligo21	ACTCTTTCCCTACACGACGCTCTTCCGATCT	NNNNNNN A NN C NNNN T NNNN G NN A NNN C NN	AGGCTATA	AGATCGGAAGAGCACACGTCTGAACTCCAGT
Oligo22	ACTCTTTCCCTACACGACGCTCTTCCGATCT	NNNNNNN A NN C NNNN T NNNN G NN A NNN C NN	GCCTCTAT	AGATCGGAAGAGCACACGTCTGAACTCCAGT
Oligo23	ACTCTTTCCCTACACGACGCTCTTCCGATCT	NNNNNNN A NN C NNNN T NNNN G NN A NNN C NN	AGGATAGG	AGATCGGAAGAGCACACGTCTGAACTCCAGT
Oligo24	ACTCTTTCCCTACACGACGCTCTTCCGATCT	NNNNNNN A NN C NNNN T NNNN G NN A NNN C NN	TCAGAGCC	AGATCGGAAGAGCACACGTCTGAACTCCAGT
Oligo25	ACTCTTTCCCTACACGACGCTCTTCCGATCT	NNNNNNN C NN A NNNN C NNNN T NN G NNN A NN	CTTCGCCT	AGATCGGAAGAGCACACGTCTGAACTCCAGT
Oligo26	ACTCTTTCCCTACACGACGCTCTTCCGATCT	NNNNNNN A NN C NNNN A NNNN C NN T NNN G NN	TAAGATTA	AGATCGGAAGAGCACACGTCTGAACTCCAGT
Oligo27	ACTCTTTCCCTACACGACGCTCTTCCGATCT	NNNNNNN G NN T NNNN C NNNN A NN C NNN A NN	ACGTCCTG	AGATCGGAAGAGCACACGTCTGAACTCCAGT
Oligo28	ACTCTTTCCCTACACGACGCTCTTCCGATCT	NNNNNNN C NN A NNNN G NNNN T NN C NNN A NN	ATGCCTAA	AGATCGGAAGAGCACACGTCTGAACTCCAGT
Oligo29	ACTCTTTCCCTACACGACGCTCTTCCGATCT	NNNNNNN G NN A NNNN T NNNN T NN C NNN T NN	GAATCTGA	AGATCGGAAGAGCACACGTCTGAACTCCAGT
Oligo30	ACTCTTTCCCTACACGACGCTCTTCCGATCT	NNNNNNN C NN T NNNN A NNNN G NN T NNN A NN	AACGTGAT	AGATCGGAAGAGCACACGTCTGAACTCCAGT
Oligo31	ACTCTTTCCCTACACGACGCTCTTCCGATCT	NNNNNNN A NN C NNNN T NNNN G NN A NNN C NN	CACTTCGA	AGATCGGAAGAGCACACGTCTGAACTCCAGT

Oligo32	ACTCTTTCCCTACACGACGCTCTTCCGATCT	NNNNNNN A NN C NNNN T NNNN G NN A NNN C NN	GCCAAGAC	AGATCGGAAGAGCACACGTCTGAACTCCAGT
Oligo33	ACTCTTTCCCTACACGACGCTCTTCCGATCT	NNNNNNN A NN C NNNN T NNNN G NN A NNN C NN	GACTAGTA	AGATCGGAAGAGCACACGTCTGAACTCCAGT
Oligo34	ACTCTTTCCCTACACGACGCTCTTCCGATCT	NNNNNNN T NN G NNNN A NNNN C NN T NNN G NN	ATTGGCTC	AGATCGGAAGAGCACACGTCTGAACTCCAGT
Oligo35	ACTCTTTCCCTACACGACGCTCTTCCGATCT	NNNNNNN G NN T NNNN C NNNN A NN G NNN A NN	GATGAATC	AGATCGGAAGAGCACACGTCTGAACTCCAGT
Oligo36	ACTCTTTCCCTACACGACGCTCTTCCGATCT	NNNNNNN C NN A NNNN G NNNN T NN C NNN T NN	AGCAGGAA	AGATCGGAAGAGCACACGTCTGAACTCCAGT
Oligo37	ACTCTTTCCCTACACGACGCTCTTCCGATCT	NNNNNNN A NN C NNNN T NNNN G NN A NNN C NN	GAGCTGAA	AGATCGGAAGAGCACACGTCTGAACTCCAGT
Oligo38	ACTCTTTCCCTACACGACGCTCTTCCGATCT	NNNNNNN C NN A NNNN C NNNN T NN G NNN A NN	AAACATCG	AGATCGGAAGAGCACACGTCTGAACTCCAGT
Oligo39	ACTCTTTCCCTACACGACGCTCTTCCGATCT	NNNNNNN A NN C NNNN A NNNN C NN T NNN G NN	GAGTTAGC	AGATCGGAAGAGCACACGTCTGAACTCCAGT
Oligo40	ACTCTTTCCCTACACGACGCTCTTCCGATCT	NNNNNNN G NN T NNNN C NNNN A NN C NNN A NN	CGAACTTA	AGATCGGAAGAGCACACGTCTGAACTCCAGT
Oligo41	ACTCTTTCCCTACACGACGCTCTTCCGATCT	NNNNNNN T NN G NNNN A NNNN C NN T NNN G NN	GATAGACA	AGATCGGAAGAGCACACGTCTGAACTCCAGT
Oligo42	ACTCTTTCCCTACACGACGCTCTTCCGATCT	NNNNNNN C NN A NNNN G NNNN T NN C NNN A NN	AAGGACAC	AGATCGGAAGAGCACACGTCTGAACTCCAGT
Oligo43	ACTCTTTCCCTACACGACGCTCTTCCGATCT	NNNNNNN G NN A NNNN T NNNN T NN C NNN T NN	GACAGTGC	AGATCGGAAGAGCACACGTCTGAACTCCAGT
Oligo44	ACTCTTTCCCTACACGACGCTCTTCCGATCT	NNNNNNN A NN C NNNN T NNNN G NN A NNN C NN	ATCATTCC	AGATCGGAAGAGCACACGTCTGAACTCCAGT
Oligo45	ACTCTTTCCCTACACGACGCTCTTCCGATCT	NNNNNNN A NN C NNNN T NNNN G NN A NNN C NN	GCCACATA	AGATCGGAAGAGCACACGTCTGAACTCCAGT
Oligo46	ACTCTTTCCCTACACGACGCTCTTCCGATCT	NNNNNNN A NN C NNNN T NNNN G NN A NNN C NN	ACCACTGT	AGATCGGAAGAGCACACGTCTGAACTCCAGT
Oligo47	ACTCTTTCCCTACACGACGCTCTTCCGATCT	NNNNNNN T NN G NNNN A NNNN C NN T NNN G NN	CTGGCATA	AGATCGGAAGAGCACACGTCTGAACTCCAGT
Oligo48	ACTCTTTCCCTACACGACGCTCTTCCGATCT	NNNNNNN G NN T NNNN C NNNN A NN G NNN A NN	ACCTCCAA	AGATCGGAAGAGCACACGTCTGAACTCCAGT
Oligo49	ACTCTTTCCCTACACGACGCTCTTCCGATCT	NNNNNNN C NN A NNNN G NNNN T NN C NNN T NN	GCGAGTAA	AGATCGGAAGAGCACACGTCTGAACTCCAGT
Oligo50	ACTCTTTCCCTACACGACGCTCTTCCGATCT	NNNNNNN A NN C NNNN T NNNN G NN A NNN C NN	ACTATGCA	AGATCGGAAGAGCACACGTCTGAACTCCAGT
Oligo51	ACTCTTTCCCTACACGACGCTCTTCCGATCT	NNNNNNN C NN A NNNN C NNNN T NN G NNN A NN	CGGATTGC	AGATCGGAAGAGCACACGTCTGAACTCCAGT
Oligo52	ACTCTTTCCCTACACGACGCTCTTCCGATCT	NNNNNNN A NN C NNNN A NNNN C NN T NNN G NN	AACTCACC	AGATCGGAAGAGCACACGTCTGAACTCCAGT
Oligo53	ACTCTTTCCCTACACGACGCTCTTCCGATCT	NNNNNNN G NN T NNNN C NNNN A NN C NNN A NN	GCTAACGA	AGATCGGAAGAGCACACGTCTGAACTCCAGT
Oligo54	ACTCTTTCCCTACACGACGCTCTTCCGATCT	NNNNNNN T NN G NNNN A NNNN C NN T NNN G NN	CAGATCTG	AGATCGGAAGAGCACACGTCTGAACTCCAGT
Oligo55	ACTCTTTCCCTACACGACGCTCTTCCGATCT	NNNNNNN C NN A NNNN G NNNN T NN C NNN A NN	ATCCTGTA	AGATCGGAAGAGCACACGTCTGAACTCCAGT
Oligo56	ACTCTTTCCCTACACGACGCTCTTCCGATCT	NNNNNNN G NN A NNNN T NNNN T NN C NNN T NN	CTGTAGCC	AGATCGGAAGAGCACACGTCTGAACTCCAGT
Oligo57	ACTCTTTCCCTACACGACGCTCTTCCGATCT	NNNNNNN A NN C NNNN T NNNN G NN A NNN C NN	GCTCGGTA	AGATCGGAAGAGCACACGTCTGAACTCCAGT
Oligo58	ACTCTTTCCCTACACGACGCTCTTCCGATCT	NNNNNNN T NN G NNNN A NNNN C NN T NNN G NN	ACACGACC	AGATCGGAAGAGCACACGTCTGAACTCCAGT
Oligo59	ACTCTTTCCCTACACGACGCTCTTCCGATCT	NNNNNNN G NN T NNNN C NNNN A NN G NNN A NN	AGTCACTA	AGATCGGAAGAGCACACGTCTGAACTCCAGT
Oligo60	ACTCTTTCCCTACACGACGCTCTTCCGATCT	NNNNNNN C NN A NNNN G NNNN T NN C NNN T NN	AACGCTTA	AGATCGGAAGAGCACACGTCTGAACTCCAGT
Oligo61	ACTCTTTCCCTACACGACGCTCTTCCGATCT	NNNNNNN A NN C NNNN T NNNN G NN A NNN C NN	GGAGAACA	AGATCGGAAGAGCACACGTCTGAACTCCAGT
Oligo62	ACTCTTTCCCTACACGACGCTCTTCCGATCT	NNNNNNN C NN A NNNN C NNNN T NN G NNN A NN	CATCAAGT	AGATCGGAAGAGCACACGTCTGAACTCCAGT
Oligo63	ACTCTTTCCCTACACGACGCTCTTCCGATCT	NNNNNNN A NN C NNNN A NNNN C NN T NNN G NN	AAGGTACA	AGATCGGAAGAGCACACGTCTGAACTCCAGT

Oligo64	ACTCTTTCCCTACACGACGCTCTTCCGATCT	NNNNNNN G NN T NNNN C NNNN A NN C NNN A NN	CGCTGATC	AGATCGGAAGAGCACACGTCTGAACTCCAGT
Oligo65	ACTCTTTCCCTACACGACGCTCTTCCGATCT	NNNNNNN T NN G NNNN A NNNN C NN T NNN G NN	GGTGCGAA	AGATCGGAAGAGCACACGTCTGAACTCCAGT
Oligo66	ACTCTTTCCCTACACGACGCTCTTCCGATCT	NNNNNNN C NN A NNNN G NNNN T NN C NNN A NN	CCTAATCC	AGATCGGAAGAGCACACGTCTGAACTCCAGT
Oligo67	ACTCTTTCCCTACACGACGCTCTTCCGATCT	NNNNNNN G NN A NNNN T NNNN T NN C NNN T NN	CTGAGCCA	AGATCGGAAGAGCACACGTCTGAACTCCAGT
Oligo68	ACTCTTTCCCTACACGACGCTCTTCCGATCT	NNNNNNN A NN C NNNN T NNNN G NN A NNN C NN	AGCCATGC	AGATCGGAAGAGCACACGTCTGAACTCCAGT
Oligo69	ACTCTTTCCCTACACGACGCTCTTCCGATCT	NNNNNNN A NN C NNNN T NNNN G NN A NNN C NN	GTACGCAA	AGATCGGAAGAGCACACGTCTGAACTCCAGT
Oligo70	ACTCTTTCCCTACACGACGCTCTTCCGATCT	NNNNNNN A NN C NNNN T NNNN G NN A NNN C NN	AGTACAAG	AGATCGGAAGAGCACACGTCTGAACTCCAGT
Oligo71	ACTCTTTCCCTACACGACGCTCTTCCGATCT	NNNNNNN T NN G NNNN A NNNN C NN T NNN G NN	ACATTGGC	AGATCGGAAGAGCACACGTCTGAACTCCAGT
Oligo72	ACTCTTTCCCTACACGACGCTCTTCCGATCT	NNNNNNN G NN T NNNN C NNNN A NN G NNN A NN	ATTGAGGA	AGATCGGAAGAGCACACGTCTGAACTCCAGT
Oligo73	ACTCTTTCCCTACACGACGCTCTTCCGATCT	NNNNNNN C NN A NNNN G NNNN T NN C NNN T NN	GTCGTAGA	AGATCGGAAGAGCACACGTCTGAACTCCAGT
Oligo74	ACTCTTTCCCTACACGACGCTCTTCCGATCT	NNNNNNN A NN C NNNN T NNNN G NN A NNN C NN	AGAGTCAA	AGATCGGAAGAGCACACGTCTGAACTCCAGT
Oligo75	ACTCTTTCCCTACACGACGCTCTTCCGATCT	NNNNNNN C NN A NNNN C NNNN T NN G NNN A NN	CCGACAAC	AGATCGGAAGAGCACACGTCTGAACTCCAGT
Oligo76	ACTCTTTCCCTACACGACGCTCTTCCGATCT	NNNNNNN A NN C NNNN A NNNN C NN T NNN G NN	ACGTATCA	AGATCGGAAGAGCACACGTCTGAACTCCAGT
Oligo77	ACTCTTTCCCTACACGACGCTCTTCCGATCT	NNNNNNN G NN T NNNN C NNNN A NN C NNN A NN	GTCTGTCA	AGATCGGAAGAGCACACGTCTGAACTCCAGT
Oligo78	ACTCTTTCCCTACACGACGCTCTTCCGATCT	NNNNNNN T NN G NNNN A NNNN C NN T NNN G NN	CTAAGGTC	AGATCGGAAGAGCACACGTCTGAACTCCAGT
Oligo79	ACTCTTTCCCTACACGACGCTCTTCCGATCT	NNNNNNN C NN A NNNN G NNNN T NN C NNN A NN	CGACACAC	AGATCGGAAGAGCACACGTCTGAACTCCAGT
Oligo80	ACTCTTTCCCTACACGACGCTCTTCCGATCT	NNNNNNN G NN A NNNN T NNNN T NN C NNN T NN	CCGTGAGA	AGATCGGAAGAGCACACGTCTGAACTCCAGT

Supplementary Table S1. DNA oligonucleotide sequences for protein indexing, and the counting barcode for the amido-bond–based method and click chemistry-based HaloTag-barcoding.

Primer	Sequence	Length
Foward1	AATGATACGGCGACCACCGAGATCT ACAC **TATAGCCT** ACACTCTTTCCCTACACGACGCTCTTCCGATCT	70
Foward2	AATGATACGGCGACCACCGAGATCT ACAC **ATAGAGGC** ACACTCTTTCCCTACACGACGCTCTTCCGATCT	70
Foward3	AATGATACGGCGACCACCGAGATCT ACAC **CCTATCCT** ACACTCTTTCCCTACACGACGCTCTTCCGATCT	70
Foward4	AATGATACGGCGACCACCGAGATCT ACAC **GGCTCTGA** ACACTCTTTCCCTACACGACGCTCTTCCGATCT	70
Foward5	AATGATACGGCGACCACCGAGATCT ACAC **AGGCGAAG** ACACTCTTTCCCTACACGACGCTCTTCCGATCT	70
Foward6	AATGATACGGCGACCACCGAGATCT ACAC **TAATCTTA** ACACTCTTTCCCTACACGACGCTCTTCCGATCT	70
Foward7	AATGATACGGCGACCACCGAGATCT ACAC **CAGGACGT** ACACTCTTTCCCTACACGACGCTCTTCCGATCT	70
Foward8	AATGATACGGCGACCACCGAGATCT ACAC **GTACTGAC** ACACTCTTTCCCTACACGACGCTCTTCCGATCT	70
Reverse1	CAAGCAGAAGACGGCATACGAGAT **CGAGTAAT** GTGACTGGAGTTCAGACGTGTGCTCTTCCGATCT	66
Reverse2	CAAGCAGAAGACGGCATACGAGAT **TCTCCGGA** GTGACTGGAGTTCAGACGTGTGCTCTTCCGATCT	66
Reverse3	CAAGCAGAAGACGGCATACGAGAT **AATGAGCG** GTGACTGGAGTTCAGACGTGTGCTCTTCCGATCT	66
Reverse4	CAAGCAGAAGACGGCATACGAGAT **GGAATCTC** GTGACTGGAGTTCAGACGTGTGCTCTTCCGATCT	66
Reverse5	CAAGCAGAAGACGGCATACGAGAT **TTCTGAAT** GTGACTGGAGTTCAGACGTGTGCTCTTCCGATCT	66
Reverse6	CAAGCAGAAGACGGCATACGAGAT **ACGAATTC** GTGACTGGAGTTCAGACGTGTGCTCTTCCGATCT	66
Reverse7	CAAGCAGAAGACGGCATACGAGAT **AGCTTCAG** GTGACTGGAGTTCAGACGTGTGCTCTTCCGATCT	66
Reverse8	CAAGCAGAAGACGGCATACGAGAT **GCGCATTA** GTGACTGGAGTTCAGACGTGTGCTCTTCCGATCT	66

Supplementary Table S2. Primer sequences for protein indexing and counting library preparation. Index sequences are underlined.

GSM_ID	FASTAQ name	Name of barcode (Supplementaly table 1)	Count	Reads	Experiments	Barcoded proteins	Proteins on beads	Reuslts	Figure	Annotation for Barcoded proteins	Annotation for Proteins on beads
GSM3473537	19_1_S1_L001_R1_001.fastq.gz	Oligo31	57	903	negative control for RRS01	HaloTag	AT2G02490		Figure 3B		
GSM3473537	19_1_S1_L001_R1_001.fastq.gz	Oligo32	178	2669	negative control for RRS02	HaloTag	AT4G23885		Figure 3B		
GSM3473537	19_1_S1_L001_R1_001.fastq.gz	Oligo33	165	2477	negative control for RRS03	HaloTag	AT3G45590		Figure 3B		
GSM3473537	19_1_S1_L001_R1_001.fastq.gz	Oligo34	161.5	2394	negative control for RRS04	HaloTag	AT4G15660		Figure 3B		
GSM3473537	19_1_S1_L001_R1_001.fastq.gz	Oligo35	359	5287	negative control for RRS05	HaloTag	AT5G07590		Figure 3B		
GSM3473537	19_1_S1_L001_R1_001.fastq.gz	Oligo36	414	6464	negative control for RRS06	HaloTag	AT3G01130		Figure 3B		
GSM3473537	19_1_S1_L001_R1_001.fastq.gz	Oligo37	104	1597	negative control for RRS07	HaloTag	AT3G26840		Figure 3B		
GSM3473537	19_1_S1_L001_R1_001.fastq.gz	Oligo38	131	2144	negative control for RRS08	HaloTag	AT1G49780		Figure 3B		
GSM3473537	19_1_S1_L001_R1_001.fastq.gz	Oligo47	86	1467	PRS01	AT4G02440	AT1G75950	Positive	Figure 3B	F-box family protein	S phase kinase-associated protein 1
GSM3473537	19_1_S1_L001_R1_001.fastq.gz	Oligo48	153	2544	PRS02	AT5G67250	AT1G75950	Positive	Figure 3B	SKP1/ASK1-interacting protein 2	S phase kinase-associated protein 1
GSM3473537	19_1_S1_L001_R1_001.fastq.gz	Oligo49	106	1541	PRS03	AT5G06950	AT1G64280	Positive	Figure 3B	bZIP transcription factor family protein	regulatory protein (NPR1)
GSM3473537	19_1_S1_L001_R1_001.fastq.gz	Oligo50	49	903	PRS04	AT4G30960	AT4G26570	Negative	Figure 3B	SOS3-interacting protein 3	calcineurin B-like 3
GSM3473537	19_1_S1_L001_R1_001.fastq.gz	Oligo51	76	1225	PRS05	AT4G01370	AT3G18690	Negative	Figure 3B	MAP kinase 4	MAP kinase substrate 1
GSM3473537	19_1_S1_L001_R1_001.fastq.gz	Oligo52	49	750	PRS06	AT2G25490	AT1G75950	Negative	Figure 3B	EIN3-binding F box protein 1	S phase kinase-associated protein 1
GSM3473537	19_1_S1_L001_R1_001.fastq.gz	Oligo53	164	2522	PRS07	AT2G35940	AT2G35940	Positive	Figure 3B	BEL1-like homeodomain 1	BEL1-like homeodomain 1
GSM3473537	19_1_S1_L001_R1_001.fastq.gz	Oligo54	74	1170	PRS08	AT5G63110	AT2G39940	Negative	Figure 3B	histone deacetylase 6	RNI-like superfamily protein
GSM3473537	19_1_S1_L001_R1_001.fastq.gz	Oligo55	43	723	RRS01	AT4G23885	AT2G02490	Negative	Figure 3B	hypothetical protein	transmembrane protein
GSM3473537	19_1_S1_L001_R1_001.fastq.gz	Oligo56	61	992	RRS02	AT2G02490	AT4G23885	Negative	Figure 3B	transmembrane protein	hypothetical protein
GSM3473537	19_1_S1_L001_R1_001.fastq.gz	Oligo57	211	3479	RRS03	AT4G15660	AT3G45590	Positive	Figure 3B	Thioredoxin superfamily protein	splicing endonuclease 1
GSM3473537	19_1_S1_L001_R1_001.fastq.gz	Oligo58	159	2568	RRS04	AT3G45590	AT4G15660	Negative	Figure 3B	splicing endonuclease 1	Thioredoxin superfamily protein
GSM3473537	19_1_S1_L001_R1_001.fastq.gz	Oligo59	195	3115	RRS05	AT3G01130	AT5G07590	Negative	Figure 3B	ATP synthase E chain	Transducin/WD40 repeat-like superfamily protein
GSM3473537	19_1_S1_L001_R1_001.fastq.gz	Oligo60	125	2016	RRS06	AT5G07590	AT3G01130	Negative	Figure 3B	Transducin/WD40 repeat-like superfamily protein	ATP synthase E chain
GSM3473537	19_1_S1_L001_R1_001.fastq.gz	Oligo61	57	976	RRS07	AT1G49780	AT3G26840	Negative	Figure 3B	plant U-box 26	Esterase/lipase/thioesterase family protein
GSM3473537	19_1_S1_L001_R1_001.fastq.gz	Oligo62	8859	126095	RRS08	AT3G26840	AT1G49780	Negative	Figure 3B	Esterase/lipase/thioesterase family protein	plant U-box 26
GSM3473537	19_1_S1_L001_R1_001.fastq.gz	Oligo71	32.5	522	negative control for PRS01	HaloTag	AT1G75950		Figure 3B		
GSM3473537	19_1_S1_L001_R1_001.fastq.gz	Oligo72	80.5	1389	negative control for PRS02	HaloTag	AT1G75950		Figure 3B		
GSM3473537	19_1_S1_L001_R1_001.fastq.gz	Oligo73	76	1173	negative control for PRS03	HaloTag	AT1G64280		Figure 3B		
GSM3473537	19_1_S1_L001_R1_001.fastq.gz	Oligo74	109	1863	negative control for PRS04	HaloTag	AT4G26570		Figure 3B		
GSM3473537	19_1_S1_L001_R1_001.fastq.gz	Oligo75	116	1789	negative control for PRS05	HaloTag	AT3G18690		Figure 3B		
GSM3473537	19_1_S1_L001_R1_001.fastq.gz	Oligo76	61	1005	negative control for PRS06	HaloTag	AT1G75950		Figure 3B		
GSM3473537	19_1_S1_L001_R1_001.fastq.gz	Oligo77	93	1724	negative control for PRS07	HaloTag	AT2G35940		Figure 3B		
GSM3473537	19_1_S1_L001_R1_001.fastq.gz	Oligo78	92	1397	negative control for PRS08	HaloTag	AT2G39940		Figure 3B		
GSM3473538	20_3_S3_L001_R1_001.fastq.gz	Oligo01	7	3630	RRS09	AT2G02490	AT3G45590	Negative*	Figure 3B	transmembrane protein	splicing endonuclease 1
GSM3473538	20_3_S3_L001_R1_001.fastq.gz	Oligo02	3	1419	RRS10	AT3G45590	AT2G02490	Negative*	Figure 3B	splicing endonuclease 1	transmembrane protein
GSM3473538	20_3_S3_L001_R1_001.fastq.gz	Oligo03	1	93	RRS11	AT5G07590	AT1G24450	Negative*	Figure 3B	Transducin/WD40 repeat-like superfamily protein	Ribonuclease III family protein
GSM3473538	20_3_S3_L001_R1_001.fastq.gz	Oligo04	10	4054	RRS12	AT3G26840	AT5G07590	Negative*	Figure 3B	Esterase/lipase/thioesterase family protein	Transducin/WD40 repeat-like superfamily protein
GSM3473538	20_3_S3_L001_R1_001.fastq.gz	Oligo17	130	48027	PRS09	AT3G18690	AT4g01370	Positive	Figure 3B	MAP kinase substrate 1	MAP kinase 4
GSM3473538	20_3_S3_L001_R1_001.fastq.gz	Oligo18	51.5	18493	PRS10	AT1G75950	AT2g25490	Positive	Figure 3B	S phase kinase-associated protein 1	EIN3-binding F box protein 1
GSM3473538	20_3_S3_L001_R1_001.fastq.gz	Oligo19	14	5742	PRS11	AT2G35940	AT2g35940	Negative*	Figure 3B	BEL1-like homeodomain 1	BEL1-like homeodomain 1
GSM3473538	20_3_S3_L001_R1_001.fastq.gz	Oligo20	3	961	PRS12	AT2G39940	AT5g63110	Negative*	Figure 3B	RNI-like superfamily protein	histone deacetylase 6
GSM3473538	20_3_S3_L001_R1_001.fastq.gz	Oligo21	33.5	11872	PRS13	AT1G14920	AT4g24210	Negative*	Figure 3B	GRAS family transcription factor family protein	F-box family protein
GSM3473538	20_3_S3_L001_R1_001.fastq.gz	Oligo22	41	14530	PRS14	AT3G62420	AT5g28770	Positive	Figure 3B	Derives from AT3G62420	bZIP transcription factor family protein
GSM3473538	20_3_S3_L001_R1_001.fastq.gz	Oligo23	8	3186	PRS15	AT2G43790	AT4g29810	Negative*	Figure 3B	MAP kinase 6	MAP kinase kinase 2
GSM3473538	20_3_S3_L001_R1_001.fastq.gz	Oligo24	1	554	PRS16	AT1G22070	AT1g64280	Negative*	Figure 3B	transcription factor TGA3	regulatory protein (NPR1)
GSM3473538	20_3_S3_L001_R1_001.fastq.gz	Oligo25	21	8316	RRS13	AT5G64000	AT2G14260	Negative*	Figure 3B	Inositol monophosphatase family protein	proline iminopeptidase
GSM3473538	20_3_S3_L001_R1_001.fastq.gz	Oligo26	0	0	RRS14	AT2G14260	AT5G64000	Negative*	Figure 3B	proline iminopeptidase	Inositol monophosphatase family protein
GSM3473538	20_3_S3_L001_R1_001.fastq.gz	Oligo27	25	9524	RRS15	AT5G62280	AT4G23885	Negative*	Figure 3B	DUF1442 family protein (DUF1442)	hypothetical protein
GSM3473538	20_3_S3_L001_R1_001.fastq.gz	Oligo28	0	0	RRS16	AT4G23885	AT5G62280	Negative*	Figure 3B	hypothetical protein	DUF1442 family protein (DUF1442)
GSM3473538	20_3_S3_L001_R1_001.fastq.gz	Oligo31	12	2762	negative control for PRS09	HaloTag	AT4g01370		Figure 3B		
GSM3473538	20_3_S3_L001_R1_001.fastq.gz	Oligo32	11	4095	negative control for PRS10	HaloTag	AT2g25490		Figure 3B		
GSM3473538	20_3_S3_L001_R1_001.fastq.gz	Oligo33	1	42	negative control for PRS11	HaloTag	AT2g35940		Figure 3B		
GSM3473538	20_3_S3_L001_R1_001.fastq.gz	Oligo34	19	7004	negative control for PRS12	HaloTag	AT5g63110		Figure 3B		
GSM3473538	20_3_S3_L001_R1_001.fastq.gz	Oligo35	0	0	negative control for PRS13	HaloTag	AT4g24210		Figure 3B		
GSM3473538	20_3_S3_L001_R1_001.fastq.gz	Oligo36	30	11188	negative control for PRS14	HaloTag	AT5g28770		Figure 3B		
GSM3473538	20_3_S3_L001_R1_001.fastq.gz	Oligo37	48	17096	negative control for PRS15	HaloTag	AT4g29810		Figure 3B		
GSM3473538	20_3_S3_L001_R1_001.fastq.gz	Oligo38	33	11898	negative control for PRS16	HaloTag	AT1g64280		Figure 3B		
GSM3473538	20_3_S3_L001_R1_001.fastq.gz	Oligo71	4	1569	negative control for RRS13	HaloTag	AT2G14260		Figure 3B		
GSM3473538	20_3_S3_L001_R1_001.fastq.gz	Oligo72	5	1952	negative control for RRS14	HaloTag	AT5G64000		Figure 3B		
GSM3473538	20_3_S3_L001_R1_001.fastq.gz	Oligo73	8	3151	negative control for RRS15	HaloTag	AT4G23885		Figure 3B		
GSM3473538	20_3_S3_L001_R1_001.fastq.gz	Oligo74	3	970	negative control for RRS16	HaloTag	AT5G62280		Figure 3B		
GSM3473538	20_3_S3_L001_R1_001.fastq.gz	Oligo75	7	3121	negative control for RRS09	HaloTag	AT3G45590		Figure 3B		
GSM3473538	20_3_S3_L001_R1_001.fastq.gz	Oligo76	3	1668	negative control for RRS10	HaloTag	AT2G02490		Figure 3B		
GSM3473538	20_3_S3_L001_R1_001.fastq.gz	Oligo77	2	719	negative control for RRS11	HaloTag	AT1G24450		Figure 3B		
GSM3473538	20_3_S3_L001_R1_001.fastq.gz	Oligo78	5	1940	negative control for RRS12	HaloTag	AT5G07590		Figure 3B		

Supplementary Table S3. Summary of the barcode pull-down assays for PPI from literature-curated interactions (Figure 3B). GSM_ID, sample ID in Gene Expression Omnibus (GEO) at the National Center for Biotechnology Information (NCBI); FASTAQ name, ID of the FASTAQ sequence file under GSE122542 in GEO (NCBI) (https://www.ncbi.nlm.nih.gov/geo/query/acc.cgi?acc=GSE122542); name of barcode, ID in Supplementary Table S1 corresponding to the barcode in protein barcode column; count, the number of barcode clusters used to determine the numbers of protein molecules; reads, the number of sequenced barcodes before clustering; experiments, class of PPI; barcoded protein and proteins on beads, the protein ID from previous reports, with A. thaliana gene IDs shown (2); results, result of the PPI assay with barcoded proteins; asterisk (*), PPI result with less than 10 reads of barcoded proteins.

GSM_ID	FASTAQ name	Name of barcode (Supplementaly table 1)	Count	Count/Total count (%)	Reads	Barcoded proteins	Annotation for Barcoded proteins	Proteins on beads
GSM3534666	45_33_S33_L001_R1_001.fastq.gz	Oligo05	539.5	1.468706613	4699	AT3G18690	MAP kinase substrate 1	AT3G62420, bZIP53
GSM3534666	45_33_S33_L001_R1_001.fastq.gz	Oligo06	182	0.495467291	1734	AT1G75950	S phase kinase-associated protein 1	AT3G62420, bZIP53
GSM3534666	45_33_S33_L001_R1_001.fastq.gz	Oligo07	151.5	0.412435685	1485	AT2G35940	BEL1-like homeodomain 1	AT3G62420, bZIP53
GSM3534666	45_33_S33_L001_R1_001.fastq.gz	Oligo08	246	0.669697547	2208	AT2G39940	RNI-like superfamily protein	AT3G62420, bZIP53
GSM3534666	45_33_S33_L001_R1_001.fastq.gz	Oligo09	278.5	0.758173849	2525.5	AT1G14920	GRAS family transcription factor family protein	AT3G62420, bZIP53
GSM3534666	45_33_S33_L001_R1_001.fastq.gz	Oligo10	538	1.464623091	4684	AT3G62420	bZIP53;basic region/leucine zipper motif 53	AT3G62420, bZIP53
GSM3534666	45_33_S33_L001_R1_001.fastq.gz	Oligo12	238	0.647918765	2291	AT2G43790	MAP kinase 6	AT3G62420, bZIP53
GSM3534666	45_33_S33_L001_R1_001.fastq.gz	Oligo13	444	1.208722402	3937	AT1G22070	TGA1A-related gene 3	AT3G62420, bZIP53
GSM3534666	45_33_S33_L001_R1_001.fastq.gz	Oligo14	854.5	2.326246155	7408	AT1G11810	F-box associated ubiquitination effector family protein	AT3G62420, bZIP53
GSM3534666	45_33_S33_L001_R1_001.fastq.gz	Oligo15	1224	3.332153649	11149	AT1G12260	VND4; NAC-domain transcription factor	AT3G62420, bZIP53
GSM3534666	45_33_S33_L001_R1_001.fastq.gz	Oligo16	980.25	2.668581385	8678	AT4G17870	PYR1; COMPONENT OF ABA RECEPTOR 11	AT3G62420, bZIP53
GSM3534666	45_33_S33_L001_R1_001.fastq.gz	Oligo39	265	0.721422154	2400	AT1G71930	VND7; NAC-domain transcription factor	AT3G62420, bZIP53
GSM3534666	45_33_S33_L001_R1_001.fastq.gz	Oligo40	1001	2.7250701	8892	AT3G62420	bZIP53; basic region/leucine zipper motif 53	AT3G62420, bZIP53
GSM3534666	45_33_S33_L001_R1_001.fastq.gz	Oligo41	418	1.137941361	3866	AT1G32640	MYC2; MYC-related transcriptional activator	AT3G62420, bZIP53
GSM3534666	45_33_S33_L001_R1_001.fastq.gz	Oligo42	1470.75	4.003892957	12696.5	AT5G28770	bZIP63; BASIC LEUCINE ZIPPER protein	AT3G62420, bZIP53
GSM3534666	45_33_S33_L001_R1_001.fastq.gz	Oligo43	427	1.16244249	3872	AT1G14200	RING; RING/U-box superfamily protein	AT3G62420, bZIP53
GSM3534666	45_33_S33_L001_R1_001.fastq.gz	Oligo44	890.5	2.424250674	8546.5	AT2G40330	PYL6; Rregulatory components of ABA receptor family proteins	AT3G62420, bZIP53
GSM3534666	45_33_S33_L001_R1_001.fastq.gz	Oligo45	1689	4.598045354	15085	AT3G08500	MYB83; putative R2R3-type MYB transcription factor	AT3G62420, bZIP53
GSM3534666	45_33_S33_L001_R1_001.fastq.gz	Oligo46	900	2.450112977	9067	AT5G62380	VND6; NAC-domain transcription factor	AT3G62420, bZIP53
GSM3534666	45_33_S33_L001_R1_001.fastq.gz	Oligo47	405	1.10255084	3682	AT4G02440	F-box family protein	AT3G62420, bZIP53
GSM3534666	45_33_S33_L001_R1_001.fastq.gz	Oligo48	815	2.218713418	7496	AT5G67250	SKP1/ASK1-interacting protein 2	AT3G62420, bZIP53
GSM3534666	45_33_S33_L001_R1_001.fastq.gz	Oligo49	1907	5.191517164	15979	AT5G06950	TGA2 bZIP transcription factor family protein	AT3G62420, bZIP53
GSM3534666	45_33_S33_L001_R1_001.fastq.gz	Oligo50	570	1.551738219	5424	AT4G30960	SOS3-interacting protein 3	AT3G62420, bZIP53
GSM3534666	45_33_S33_L001_R1_001.fastq.gz	Oligo51	1173	3.193313914	10134	AT4G01370	MAP kinase 4	AT3G62420, bZIP53
GSM3534666	45_33_S33_L001_R1_001.fastq.gz	Oligo52	536	1.459178395	5354	AT5G07590	Transducin/WD40 repeat-like superfamily protein	AT3G62420, bZIP53
GSM3534666	45_33_S33_L001_R1_001.fastq.gz	Oligo53	506	1.377507963	4692	AT1G49780	plant U-box 26	AT3G62420, bZIP53
GSM3534666	45_33_S33_L001_R1_001.fastq.gz	Oligo54	426	1.159720143	3711	AT3G26840	Esterase/lipase/thioesterase family protein	AT3G62420, bZIP53
GSM3534666	45_33_S33_L001_R1_001.fastq.gz	Oligo55	1222	3.326708954	11129	AT4G23885	hypothetical protein	AT3G62420, bZIP53
GSM3534666	45_33_S33_L001_R1_001.fastq.gz	Oligo56	429.5	1.16924836	4250	AT2G02490	transmembrane protein	AT3G62420, bZIP53
GSM3534666	45_33_S33_L001_R1_001.fastq.gz	Oligo57	829	2.256826287	7441	AT4G15660	Thioredoxin superfamily protein	AT3G62420, bZIP53
GSM3534666	45_33_S33_L001_R1_001.fastq.gz	Oligo58	532	1.448289004	4916	AT3G45590	splicing endonuclease 1	AT3G62420, bZIP53
GSM3534666	45_33_S33_L001_R1_001.fastq.gz	Oligo59	518	1.410176136	4589	AT3G01130	ATP synthase E chain	AT3G62420, bZIP53
GSM3534666	45_33_S33_L001_R1_001.fastq.gz	Oligo60	528	1.437399613	4830	AT5G07590	Transducin/WD40 repeat-like superfamily protein	AT3G62420, bZIP53
GSM3534666	45_33_S33_L001_R1_001.fastq.gz	Oligo61	499	1.358451529	4712	AT1G49780	plant U-box 26	AT3G62420, bZIP53
GSM3534666	45_33_S33_L001_R1_001.fastq.gz	Oligo62	727	1.979146816	6783	AT3G26840	Esterase/lipase/thioesterase family protein	AT3G62420, bZIP53
GSM3534666	45_33_S33_L001_R1_001.fastq.gz	Oligo63	367	0.999101625	3289	AT4G24210	F-box family protein	AT3G62420, bZIP53
GSM3534666	45_33_S33_L001_R1_001.fastq.gz	Oligo64	968.5	2.636593798	8819	AT5G28770	bZIP63; BASIC LEUCINE ZIPPER protein	AT3G62420, bZIP53
GSM3534666	45_33_S33_L001_R1_001.fastq.gz	Oligo65	441	1.200555359	3807	AT4G29810	MAP kinase kinase 2	AT3G62420, bZIP53
GSM3534666	45_33_S33_L001_R1_001.fastq.gz	Oligo66	685	1.864808211	6441	AT1G64280	regulatory protein (NPR1)	AT3G62420, bZIP53
GSM3534666	45_33_S33_L001_R1_001.fastq.gz	Oligo67	367	0.999101625	3508	AT1G76580	Squamosa promoter-binding protein-like transcription factor family protein	AT3G62420, bZIP53
GSM3534666	45_33_S33_L001_R1_001.fastq.gz	Oligo68	343.5	0.935126453	3204.5	AT1G07030	Mitochondrial substrate carrier family protein	AT3G62420, bZIP53
GSM3534666	45_33_S33_L001_R1_001.fastq.gz	Oligo69	838	2.281327417	7187	AT4G34840	Phosphorylase superfamily protein	AT3G62420, bZIP53
GSM3534666	45_33_S33_L001_R1_001.fastq.gz	Oligo70	303	0.824871369	2815	AT5G64000	Inositol monophosphatase family protein	AT3G62420, bZIP53
GSM3534666	45_33_S33_L001_R1_001.fastq.gz	Oligo71	790.5	2.152015899	7524.5	HaloTag	negative control	AT3G62420, bZIP53
GSM3534666	45_33_S33_L001_R1_001.fastq.gz	Oligo72	417	1.135219013	3881	HaloTag	negative control	AT3G62420, bZIP53
GSM3534666	45_33_S33_L001_R1_001.fastq.gz	Oligo73	1097.5	2.987776659	10210	HaloTag	negative control	AT3G62420, bZIP53
GSM3534666	45_33_S33_L001_R1_001.fastq.gz	Oligo74	897	2.441945934	8368	HaloTag	negative control	AT3G62420, bZIP53
GSM3534666	45_33_S33_L001_R1_001.fastq.gz	Oligo75	1559	4.244140146	15435	HaloTag	negative control	AT3G62420, bZIP53
GSM3534666	45_33_S33_L001_R1_001.fastq.gz	Oligo76	924	2.515449323	8880	HaloTag	negative control	AT3G62420, bZIP53
GSM3534666	45_33_S33_L001_R1_001.fastq.gz	Oligo77	1423	3.873900852	14180	HaloTag	negative control	AT3G62420, bZIP53
GSM3534666	45_33_S33_L001_R1_001.fastq.gz	Oligo78	952	2.591675061	9152	HaloTag	negative control	AT3G62420, bZIP53
		Total	36733	100				
	Negative control average (Oligo71-78)			2.742765361				

Supplementary Table S4. Summary of a high-throughput assay with a prepared 51 barcoded protein mixture shown in Supplementary Figure 4. GSM_ID, sample ID in Gene Expression Omnibus (GEO) at the National Center for Biotechnology Information (NCBI); FASTAQ name, the ID of the FASTAQ sequence file under GSE122542 in GEO (NCBI) (https://www.ncbi.nlm.nih.gov/geo/query/acc.cgi?acc=GSE122542). name of barcode, ID in Supplementary Table 1 corresponding to the barcode in protein barcode column; count, the number of barcode clusters used to determine the numbers of protein molecules; count/total count (%), the ratio of each barcoded protein to total reads of barcoded proteins (36,733 read counts); reads, the number of sequenced barcodes before clustering. The 43 barcoded proteins and 8 HaloTag-only proteins were barcoded with Oligo05–10, 12–16, and 39–78 (Supplementary Table S1). Barcoded protein and proteins on beads, the ID of the protein from previous reports, with A. thaliana gene IDs shown (2).

Dilution	Halo-NL (qPCR)	Halo-NL (reads)	Halo-NL-JUN (qPCR)	Halo-NL-JUN (reads)	Halo-NL-FOS (qPCR)	Halo-NL-FOS (reads)	Bkg (reads)
$x10^4$	98400	8138	36000	7946	12000	4261	5.9
$x10^5$	9840	5064	3600	1860	1200	340	3.6
$x10^6$	984	501	360	167	120	32	5.3
$x10^7$	98.4	28	36	1	12	21	1.6
$x10^8$	9.84	4	3.6	0	1.2	8	1.2
$x10^9$	0.984	1	0.36	0	0.12	6	2.0

3SD+average=9.2

Supplementary Table S5. Summary of the dynamic range of protein assays by DNA barcode. Barcoded HaloTag-protein complexes were prepared using different dilution series based on quantitative polymerase chain reaction (qPCR) analysis, and simultaneously assayed by DNA barcode sequencing and the luciferase assay (Figure 2D). Halo-NL (qPCR), Halo-NL-JUN (qPCR), and Halo-NL-FOS (qPCR) indicate the number of molecules of each barcoded protein identified by qPCR. Halo-NL (reads), Halo-NL-JUN (reads), and Halo-NL-FOS (reads) indicate the number of unique molecules of each barcoded protein identified by barcode counting. Bkg (reads), the number of unique reads from DNA barcode sequence that was not used in the assay.

GSM_ID	FASTAQ name	Name of barcode (Supplementaly table 1)	Count	Reads	Experiments	Figure
GSM3473519	01_41_S21_L001_R1_001.fastq.gz	Oligo01	8138	8865	10^4 dilution of NL-Oligo01	Figure 2D (blue)
GSM3473519	01_41_S21_L001_R1_001.fastq.gz	Oligo03	1	1	10^4 dilution of NL-Oligo01	
GSM3473519	01_41_S21_L001_R1_001.fastq.gz	Oligo04	11	12	10^4 dilution of NL-Oligo01	
GSM3473519	01_41_S21_L001_R1_001.fastq.gz	Oligo05	4	4	10^4 dilution of NL-Oligo01	
GSM3473519	01_41_S21_L001_R1_001.fastq.gz	Oligo06	0	0	10^4 dilution of NL-Oligo01	
GSM3473520	02_42_S22_L001_R1_001.fastq.gz	Oligo01	5064	102606	10^5 dilution of NL-Oligo01	Figure 2D (blue)
GSM3473520	02_42_S22_L001_R1_001.fastq.gz	Oligo03	0	1	10^5 dilution of NL-Oligo01	
GSM3473520	02_42_S22_L001_R1_001.fastq.gz	Oligo04	10	172	10^5 dilution of NL-Oligo01	
GSM3473520	02_42_S22_L001_R1_001.fastq.gz	Oligo05	2	28	10^5 dilution of NL-Oligo01	
GSM3473520	02_42_S22_L001_R1_001.fastq.gz	Oligo06	3	93	10^5 dilution of NL-Oligo01	
GSM3473521	03_43_S23_L001_R1_001.fastq.gz	Oligo01	501	41522	10^6 dilution of NL-Oligo01	Figure 2D (blue)
GSM3473521	03_43_S23_L001_R1_001.fastq.gz	Oligo03	3	179	10^6 dilution of NL-Oligo01	
GSM3473521	03_43_S23_L001_R1_001.fastq.gz	Oligo04	4	493	10^6 dilution of NL-Oligo01	
GSM3473521	03_43_S23_L001_R1_001.fastq.gz	Oligo05	11	1045	10^6 dilution of NL-Oligo01	
GSM3473521	03_43_S23_L001_R1_001.fastq.gz	Oligo06	3	180	10^6 dilution of NL-Oligo01	
GSM3473522	04_44_S24_L001_R1_001.fastq.gz	Oligo01	28	26319	10^7 dilution of NL-Oligo01	Figure 2D (blue)
GSM3473522	04_44_S24_L001_R1_001.fastq.gz	Oligo03	0	0	10^7 dilution of NL-Oligo01	
GSM3473522	04_44_S24_L001_R1_001.fastq.gz	Oligo04	0	0	10^7 dilution of NL-Oligo01	
GSM3473522	04_44_S24_L001_R1_001.fastq.gz	Oligo05	0	0	10^7 dilution of NL-Oligo01	
GSM3473522	04_44_S24_L001_R1_001.fastq.gz	Oligo06	0	0	10^7 dilution of NL-Oligo01	
GSM3473523	05_49_S29_L001_R1_001.fastq.gz	Oligo01	4	12153	10^8 dilution of NL-Oligo01	Figure 2D (blue)
GSM3473523	05_49_S29_L001_R1_001.fastq.gz	Oligo03	0	0	10^8 dilution of NL-Oligo01	
GSM3473523	05_49_S29_L001_R1_001.fastq.gz	Oligo04	4	4105	10^8 dilution of NL-Oligo01	
GSM3473523	05_49_S29_L001_R1_001.fastq.gz	Oligo05	1	3479	10^8 dilution of NL-Oligo01	
GSM3473523	05_49_S29_L001_R1_001.fastq.gz	Oligo06	0	0	10^8 dilution of NL-Oligo01	
GSM3473524	06_50_S30_L001_R1_001.fastq.gz	Oligo01	1	5571	10^9 dilution of NL-Oligo01	Figure 2D (blue)
GSM3473524	06_50_S30_L001_R1_001.fastq.gz	Oligo03	2	4876	10^9 dilution of NL-Oligo01	
GSM3473524	06_50_S30_L001_R1_001.fastq.gz	Oligo04	1	3352	10^9 dilution of NL-Oligo01	
GSM3473524	06_50_S30_L001_R1_001.fastq.gz	Oligo05	2	14402	10^9 dilution of NL-Oligo01	
GSM3473524	06_50_S30_L001_R1_001.fastq.gz	Oligo06	0	0	10^9 dilution of NL-Oligo01	
GSM3473525	07_44_S44_L001_R1_001.fastq.gz	Oligo01	9	424	10^4 dilution of NL-FOS-Oligo06	
GSM3473525	07_44_S44_L001_R1_001.fastq.gz	Oligo03	7.5	413.5	10^4 dilution of NL-FOS-Oligo06	
GSM3473525	07_44_S44_L001_R1_001.fastq.gz	Oligo04	5	178	10^4 dilution of NL-FOS-Oligo06	
GSM3473525	07_44_S44_L001_R1_001.fastq.gz	Oligo05	7	339	10^4 dilution of NL-FOS-Oligo06	
GSM3473525	07_44_S44_L001_R1_001.fastq.gz	Oligo06	4261	178419	10^4 dilution of NL-FOS-Oligo06	Figure 2D (red)
GSM3473526	08_45_S45_L001_R1_001.fastq.gz	Oligo01	4	1235	10^5 dilution of NL-FOS-Oligo06	
GSM3473526	08_45_S45_L001_R1_001.fastq.gz	Oligo03	2	1082	10^5 dilution of NL-FOS-Oligo06	
GSM3473526	08_45_S45_L001_R1_001.fastq.gz	Oligo04	8	3924	10^5 dilution of NL-FOS-Oligo06	
GSM3473526	08_45_S45_L001_R1_001.fastq.gz	Oligo05	8	3303	10^5 dilution of NL-FOS-Oligo06	
GSM3473526	08_45_S45_L001_R1_001.fastq.gz	Oligo06	340	172203	10^5 dilution of NL-FOS-Oligo06	Figure 2D (red)
GSM3473527	09_46_S46_L001_R1_001.fastq.gz	Oligo01	36	1707	10^6 dilution of NL-FOS-Oligo06	
GSM3473527	09_46_S46_L001_R1_001.fastq.gz	Oligo03	3	167	10^6 dilution of NL-FOS-Oligo06	
GSM3473527	09_46_S46_L001_R1_001.fastq.gz	Oligo04	15	651	10^6 dilution of NL-FOS-Oligo06	
GSM3473527	09_46_S46_L001_R1_001.fastq.gz	Oligo05	36	1345	10^6 dilution of NL-FOS-Oligo06	
GSM3473527	09_46_S46_L001_R1_001.fastq.gz	Oligo06	32	1543	10^6 dilution of NL-FOS-Oligo06	Figure 2D (red)
GSM3473528	10_47_S47_L001_R1_001.fastq.gz	Oligo01	14	39076	10^7 dilution of NL-FOS-Oligo06	
GSM3473528	10_47_S47_L001_R1_001.fastq.gz	Oligo03	2	8478	10^7 dilution of NL-FOS-Oligo06	
GSM3473528	10_47_S47_L001_R1_001.fastq.gz	Oligo04	1	3580	10^7 dilution of NL-FOS-Oligo06	
GSM3473528	10_47_S47_L001_R1_001.fastq.gz	Oligo05	9	32256	10^7 dilution of NL-FOS-Oligo06	
GSM3473528	10_47_S47_L001_R1_001.fastq.gz	Oligo06	21	70289	10^7 dilution of NL-FOS-Oligo06	Figure 2D (red)
GSM3473529	11_48_S48_L001_R1_001.fastq.gz	Oligo01	2	2991	10^8 dilution of NL-FOS-Oligo06	
GSM3473529	11_48_S48_L001_R1_001.fastq.gz	Oligo03	0	0	10^8 dilution of NL-FOS-Oligo06	
GSM3473529	11_48_S48_L001_R1_001.fastq.gz	Oligo04	3	2545	10^8 dilution of NL-FOS-Oligo06	
GSM3473529	11_48_S48_L001_R1_001.fastq.gz	Oligo05	5	9178	10^8 dilution of NL-FOS-Oligo06	
GSM3473529	11_48_S48_L001_R1_001.fastq.gz	Oligo06	8	11559	10^8 dilution of NL-FOS-Oligo06	Figure 2D (red)
GSM3473530	12_49_S49_L001_R1_001.fastq.gz	Oligo01	3	3558	10^9 dilution of NL-FOS-Oligo06	
GSM3473530	12_49_S49_L001_R1_001.fastq.gz	Oligo03	4	4975	10^9 dilution of NL-FOS-Oligo06	
GSM3473530	12_49_S49_L001_R1_001.fastq.gz	Oligo04	20	14935	10^9 dilution of NL-FOS-Oligo06	
GSM3473530	12_49_S49_L001_R1_001.fastq.gz	Oligo05	9	9001	10^9 dilution of NL-FOS-Oligo06	
GSM3473530	12_49_S49_L001_R1_001.fastq.gz	Oligo06	6	10221	10^9 dilution of NL-FOS-Oligo06	Figure 2D (red)
GSM3473531	13_45_S25_L001_R1_001.fastq.gz	Oligo01	4	6	10^4 dilution of NL-JUN-Oligo03	
GSM3473531	13_45_S25_L001_R1_001.fastq.gz	Oligo03	7946	9997	10^4 dilution of NL-JUN-Oligo03	Figure 2D (green)
GSM3473531	13_45_S25_L001_R1_001.fastq.gz	Oligo04	0	0	10^4 dilution of NL-JUN-Oligo03	
GSM3473531	13_45_S25_L001_R1_001.fastq.gz	Oligo05	0	0	10^4 dilution of NL-JUN-Oligo03	
GSM3473531	13_45_S25_L001_R1_001.fastq.gz	Oligo06	32	38	10^4 dilution of NL-JUN-Oligo03	
GSM3473532	14_46_S26_L001_R1_001.fastq.gz	Oligo01	1	117	10^5 dilution of NL-JUN-Oligo03	
GSM3473532	14_46_S26_L001_R1_001.fastq.gz	Oligo03	1860	231957	10^5 dilution of NL-JUN-Oligo03	Figure 2D (green)
GSM3473532	14_46_S26_L001_R1_001.fastq.gz	Oligo04	0	0	10^5 dilution of NL-JUN-Oligo03	
GSM3473532	14_46_S26_L001_R1_001.fastq.gz	Oligo05	0	0	10^5 dilution of NL-JUN-Oligo03	
GSM3473532	14_46_S26_L001_R1_001.fastq.gz	Oligo06	13	1704	10^5 dilution of NL-JUN-Oligo03	
GSM3473533	15_47_S27_L001_R1_001.fastq.gz	Oligo01	5	3083	10^6 dilution of NL-JUN-Oligo03	
GSM3473533	15_47_S27_L001_R1_001.fastq.gz	Oligo03	167	165057	10^6 dilution of NL-JUN-Oligo03	Figure 2D (green)
GSM3473533	15_47_S27_L001_R1_001.fastq.gz	Oligo04	0	0	10^6 dilution of NL-JUN-Oligo03	
GSM3473533	15_47_S27_L001_R1_001.fastq.gz	Oligo05	0	0	10^6 dilution of NL-JUN-Oligo03	
GSM3473533	15_47_S27_L001_R1_001.fastq.gz	Oligo06	3	3348	10^6 dilution of NL-JUN-Oligo03	
GSM3473534	16_52_S32_L001_R1_001.fastq.gz	Oligo01	0	0	10^7 dilution of NL-JUN-Oligo03	
GSM3473534	16_52_S32_L001_R1_001.fastq.gz	Oligo03	1	2531	10^7 dilution of NL-JUN-Oligo03	Figure 2D (green)

GSM3473534	16_52_S32_L001_R1_001.fastq.gz	Oligo04	0	0	10^7 dilution of NL-JUN-Oligo03	
GSM3473534	16_52_S32_L001_R1_001.fastq.gz	Oligo05	0	0	10^7 dilution of NL-JUN-Oligo03	
GSM3473534	16_52_S32_L001_R1_001.fastq.gz	Oligo06	0	0	10^7 dilution of NL-JUN-Oligo03	
GSM3473535	17_53_S33_L001_R1_001.fastq.gz	Oligo01	0	0	10^8 dilution of NL-JUN-Oligo03	
GSM3473535	17_53_S33_L001_R1_001.fastq.gz	Oligo03	0	0	10^8 dilution of NL-JUN-Oligo03	Figure 2D (green)
GSM3473535	17_53_S33_L001_R1_001.fastq.gz	Oligo04	0	0	10^8 dilution of NL-JUN-Oligo03	
GSM3473535	17_53_S33_L001_R1_001.fastq.gz	Oligo05	0	0	10^8 dilution of NL-JUN-Oligo03	
GSM3473535	17_53_S33_L001_R1_001.fastq.gz	Oligo06	0	0	10^8 dilution of NL-JUN-Oligo03	
GSM3473536	18_54_S34_L001_R1_001.fastq.gz	Oligo01	0	0	10^9 dilution of NL-JUN-Oligo03	
GSM3473536	18_54_S34_L001_R1_001.fastq.gz	Oligo03	0	0	10^9 dilution of NL-JUN-Oligo03	Figure 2D (green)
GSM3473536	18_54_S34_L001_R1_001.fastq.gz	Oligo04	0	0	10^9 dilution of NL-JUN-Oligo03	
GSM3473536	18_54_S34_L001_R1_001.fastq.gz	Oligo05	0	0	10^9 dilution of NL-JUN-Oligo03	
GSM3473536	18_54_S34_L001_R1_001.fastq.gz	Oligo06	0	0	10^9 dilution of NL-JUN-Oligo03	

Supplementary Table S6. Summary of the dynamic range of HaloTag barcode assay shown in Figure 2D. GSM_ID, the sample ID in GEO (NCBI); FASTAQ name, the ID of the FASTAQ sequence file under GSE122542 in GEO (NCBI) (https://www.ncbi.nlm.nih.gov/geo/query/acc.cgi?acc=GSE122542); name of barcode, the name of barcode in Supplementary Table S1; count, the number of barcode clusters used to determine the numbers of protein molecules; reads, the number of sequenced barcodes before clustering; experiments, the type and dilution of protein used in the HaloTag barcoding assay; figure, correspondence with a data point in Figure 2D.

		Count								Reads									Barcoded proteins			
GSM_ID	FASTAQ name	Oligo59	Oligo60	Oligo61	Oligo62	Oligo71	Oligo72	Oligo73	Oligo74	Oligo59	Oligo60	Oligo61	Oligo62	Oligo71	Oligo72	Oligo73	Oligo74	Experiments	Barcoded DSG3 (Oligo59-62)	Barcoded HaloTag (Oligo71-74)	Proteins on beads	Figure
GSM3473539	21_1_S1_L001_R1_001.fastq.gz	4	17	612	170	2	7	2	3	766	5661	176759	51409	842	944	1483	1656	IP01	540 pM barcoded protins (DSG3)	515 pM barcoded protins (HaloTag)	10^{4} dilution antiDSG3 antibody	Figure 3C (blue)
GSM3473540	22_2_S2_L001_R1_001.fastq.gz	26	10	2851	1162	2	6	7	5	2491	951	290371	126937	263	195	626	613	IP02	216 pM barcoded protins (DSG3)	206 pM barcoded protins (HaloTag)	10^{4} dilution antiDSG3 antibody	Figure 3C (red)
GSM3473541	23_3_S3_L001_R1_001.fastq.gz	263	146	4159.5	2214	78	69	45	76	22788	11756	369517	210770	7353	5099	3794	6710	IP03	108 pM barcoded protins (DSG3)	103 pM barcoded protins (HaloTag)	10^{4} dilution antiDSG3 antibody	Figure 3C (green)
GSM3473542	24_5_S5_L001_R1_001.fastq.gz	4	16	143	185	2	13	1	1	2808	12318	106194	164366	6635	2087	916	773	IP04	540 pM barcoded protins (DSG3)	515 pM barcoded protins (HaloTag)	10^{8} dilution antiDSG3 antibody	Figure 3C (blue)
GSM3473543	25_6_S6_L001_R1_001.fastq.gz	15	5	641	32	4	14	2	3	11436	882	329028	15500	4456	3234	183	911	IP05	216 pM barcoded protins (DSG3)	206 pM barcoded protins (HaloTag)	10^{8} dilution antiDSG3 antibody	Figure 3C (red)
GSM3473544	26_7_S7_L001_R1_001.fastq.gz	138	149	1520	1010	38	22	34	61	23426	26421	255328	182792	5863	3929	6558	9965	IP06	108 pM barcoded protins (DSG3)	103 pM barcoded protins (HaloTag)	10^{8} dilution antiDSG3 antibody	Figure 3C (green)
GSM3473545	27_9_S9_L001_R1_001.fastq.gz	8	11	172	28	1	2	0	0	11532	13673	195948	27621	998	1749	0	0	IP07	540 pM barcoded protins (DSG3)	515 pM barcoded protins (HaloTag)	10^{12} dilution antiDSG3 antibody	Figure 3C (blue)
GSM3473546	28_10_S10_L001_R1_001.fastq.gz	3	24	63	7	0	7	1	4	3916	22192	77751	14276	0	15	1495	4518	IP08	216 pM barcoded protins (DSG3)	206 pM barcoded protins (HaloTag)	10^{12} dilution antiDSG3 antibody	Figure 3C (red)
GSM3473547	29_11_S11_L001_R1_001.fastq.gz	18	9	200	79.5	7	10	4	14	16427	7559	175960	67484	5644	3428	4047	12052	IP09	108 pM barcoded protins (DSG3)	103 pM barcoded protins (HaloTag)	10^{12} dilution antiDSG3 antibody	Figure 3C (green)
GSM3473548	30_13_S13_L001_R1_001.fastq.gz	9	13	62	33	0	9	0	1	11090	17949	76706	31579	0	14	0	1666	IP10	540 pM barcoded protins (DSG3)	515 pM barcoded protins (HaloTag)	10^{18} dilution antiDSG3 antibody	Figure 3C (blue)
GSM3473549	31_14_S14_L001_R1_001.fastq.gz	10	7	74	14.5	1	18.5	0	1	11592	7055	84888	20919.5	461	3375.5	0	1890	IP11	216 pM barcoded protins (DSG3)	206 pM barcoded protins (HaloTag)	10^{18} dilution antiDSG3 antibody	Figure 3C (red)
GSM3473550	32_15_S15_L001_R1_001.fastq.gz	16	50	432	1119	53	13	23	28	4814	13113	115824	323680	11332	2641	5448	7285	IP12	108 pM barcoded protins (DSG3)	103 pM barcoded protins (HaloTag)	10^{18} dilution antiDSG3 antibody	Figure 3C (green)
GSM3473551	33_17_S17_L001_R1_001.fastq.gz	9	7	34694	76	1	3	0	6	145	105	522387	1114	5	33	0	68	negative control for IP01	540 pM barcoded protins (DSG3)	515 pM barcoded protins (HaloTag)	10^{4} dilution human serum	
GSM3473552	34_18_S18_L001_R1_001.fastq.gz	5	13	7363	199	2	0	3	6	372	778	520986	15309	209	0	165	527	negative control for IP02	216 pM barcoded protins (DSG3)	206 pM barcoded protins (HaloTag)	10^{4} dilution human serum	
GSM3473553	35_19_S19_L001_R1_001.fastq.gz	173	363	2099.5	908	31	24	38	87	22729	46251	291439	139374	4543	2690	4484	11330	negative control for IP03	108 pM barcoded protins (DSG3)	103 pM barcoded protins (HaloTag)	10^{4} dilution human serum	
GSM3473554	36_21_S21_L001_R1_001.fastq.gz	12	14	84.5	187	1	12	1	2	9860	16135	60642	180702	514	4124	3	1263	negative control for IP04	540 pM barcoded protins (DSG3)	515 pM barcoded protins (HaloTag)	10^{8} dilution human serum	
GSM3473555	37_22_S22_L001_R1_001.fastq.gz	89	9	738	413	0	6	1	2	36363	2407	256276	159973	0	7	208	329	negative control for IP05	216 pM barcoded protins (DSG3)	206 pM barcoded protins (HaloTag)	10^{8} dilution human serum	
GSM3473556	38_23_S23_L001_R1_001.fastq.gz	30	18	441	115	11	3	5	9	15637	11782	238261	67920	7631	994	3778	5116	negative control for IP06	108 pM barcoded protins (DSG3)	103 pM barcoded protins (HaloTag)	10^{8} dilution human serum	
GSM3473557	39_25_S25_L001_R1_001.fastq.gz	27	8	335	84	2	123	2	4	544	76	7116	1678	49	435	48	21	negative control for IP07	540 pM barcoded protins (DSG3)	515 pM barcoded protins (HaloTag)	10^{12} dilution human serum	
GSM3473558	40_26_S26_L001_R1_001.fastq.gz	41	166	1899	2935	10	9	20	25	3650	15035	170628	284674	575	365	1536	2135	negative control for IP08	216 pM barcoded protins (DSG3)	206 pM barcoded protins (HaloTag)	10^{12} dilution human serum	
GSM3473559	41_27_S27_L001_R1_001.fastq.gz	70	233	5514.5	834	45	42	50	66	3542	11755	284810	45612	2476	1876	2461	3299	negative control for IP09	108 pM barcoded protins (DSG3)	103 pM barcoded protins (HaloTag)	10^{12} dilution human serum	
GSM3473560	42_29_S29_L001_R1_001.fastq.gz	11	0	1742	128	103	0	1	0	2428	0	396753	30489	23813	0	86	0	negative control for IP10	540 pM barcoded protins (DSG3)	515 pM barcoded protins (HaloTag)	10^{18} dilution human serum	
GSM3473561	43_30_S30_L001_R1_001.fastq.gz	34	24	994	477	3	1	6	5	11822	6286	315868	168342	987	340	1745	786	negative control for IP11	216 pM barcoded protins (DSG3)	206 pM barcoded protins (HaloTag)	10^{18} dilution human serum	
GSM3473562	44_31_S31_L001_R1_001.fastq.gz	902	568	1950.5	327	22	13	35	44	96128	60858	207425	36800	2427	1220	3439	4584	negative control for IP12	108 pM barcoded protins (DSG3)	103 pM barcoded protins (HaloTag)	10^{18} dilution human serum	

Supplementary Table S7. Summary of the DSG3 barcode immunoprecipitation experiment shown in Figure 3C. GSM_ID, the sample ID in GEO (NCBI); FASTAQ name, the ID of the FASTAQ sequence file under GSE122542 in GEO (NCBI) (https://www.ncbi.nlm.nih.gov/geo/query/acc.cgi?acc=GSE122542); count, the number of barcode clusters used to determine the numbers of protein molecules; reads, the number of sequenced barcodes before clustering. The DSG3 and HaloTag-only proteins were barcoded with Oligo59–62 and Oligo71–74 (Supplementary Table S1). Barcoded proteins, concentration of each barcoded protein, as determined by qPCR; proteins on beads, the types of antibody captured by protein G beads; figure, correspondence with a data point in Figure 3C.

GSM_ID	FASTAQ name	Count								Reads								Barcoded proteins		Antibody on proteinG beads	Figure
		Oligo59	Oligo60	Oligo61	Oligo62	Oligo75	Oligo76	Oligo77	Oligo78	Oligo59	Oligo60	Oligo61	Oligo62	Oligo75	Oligo76	Oligo77	Oligo78				
SM4041477	46_25_S22_L001_R1_001.fastq.gz	2287.5	213	91	3099					71703.5	6029	2829	112127					Barcoded DSG3 (Oligo59-62)		10^4 dilution serum from H1	Figure 4A, H1
SM4041478	47_29_S26_L001_R1_001.fastq.gz					1096	857	668	1007					62736	52149	38841	58586		Barcoded HaloTag (Oligo75-78)	10^4 dilution serum from H1	Figure 4A, H1
SM4041479	48_10_S7_L001_R1_001.fastq.gz	1326	186	8	5948					37636	5516	175	193857					Barcoded DSG3 (Oligo59-62)		10^6 dilution serum from H2	Figure 4A, H2
SM4041480	49_14_S11_L001_R1_001.fastq.gz					1597	867	782	1459					90327	50000	45253	80691		Barcoded HaloTag (Oligo75-78)	10^6 dilution serum from H2	Figure 4A, H2
SM4041481	50_1_S30_L001_R1_001.fastq.gz	1623.5	265	952.5	43528.5					11033	1655	6302	310319					Barcoded DSG3 (Oligo59-62)		10^4 dilution serum from D1	Figure 4A, D1
SM4041482	51_5_S2_L001_R1_001.fastq.gz					1740	1247	966	1433					76026	55712	43381	61814		Barcoded HaloTag (Oligo75-78)	10^4 dilution serum from D1	Figure 4A, D1
SM4041483	52_18_S15_L001_R1_001.fastq.gz	95670.33	182.5	205	2004.167					568572	859	1023	10628					Barcoded DSG3 (Oligo59-62)		10^6 dilution serum from D2	Figure 4A, D2
SM4041484	53_22_S19_L001_R1_001.fastq.gz					2202.5	1024.5	850	1453					137829	67138	56005	89900		Barcoded HaloTag (Oligo75-78)	10^6 dilution serum from D2	Figure 4A, D2

Supplementary Table S8. Summary of the PV patient's autoimmune antibody detection with barcoded Desmoglein 3 (DSG3) in Figure 4A. 'FASTAQ name' column indicates the ID of the FASTAQ sequence file under GSE122542 in GEO in NCBI (https://www.ncbi.nlm.nih.gov/geo/query/acc.cgi?acc=GSE122542). 'Count' column indicates number of barcode cluster to determine the numbers of protein molecules. 'reads' column indicates number of sequenced barcode before clustering. The DSG3 and HaloTag only proteins were barcoded with Oligo59-62 and Oligo75-78 in Supplementary Table S1.'Barcoded proteins' column indicates a types of each barcoded proteins with barcode ID in Supplementary Tabe S1. 'Antibody on proteinG beads' column indicates dilution ratio and types of antibody captured by protein G beads. 'Figure' column indicate correspondence to the data point of Figure 4A.
